# Three SRA-Domain Methylcytosine-Binding Proteins Cooperate to Maintain Global CpG Methylation and Epigenetic Silencing in Arabidopsis

**DOI:** 10.1371/journal.pgen.1000156

**Published:** 2008-08-15

**Authors:** Hye Ryun Woo, Travis A. Dittmer, Eric J. Richards

**Affiliations:** Department of Biology, Washington University in St. Louis, St. Louis, Missouri, United States of America; National Institute of Genetics, Japan

## Abstract

Methylcytosine-binding proteins decipher the epigenetic information encoded by DNA methylation and provide a link between DNA methylation, modification of chromatin structure, and gene silencing. *VARIANT IN METHYLATION 1* (*VIM1*) encodes an SRA (SET- and RING-associated) domain methylcytosine-binding protein in *Arabidopsis thaliana*, and loss of *VIM1* function causes centromere DNA hypomethylation and centromeric heterochromatin decondensation in interphase. In the Arabidopsis genome, there are five *VIM* genes that share very high sequence similarity and encode proteins containing a PHD domain, two RING domains, and an SRA domain. To gain further insight into the function and potential redundancy among the VIM proteins, we investigated strains combining different *vim* mutations and transgenic *vim* knock-down lines that down-regulate multiple *VIM* family genes. The *vim1 vim3* double mutant and the transgenic *vim* knock-down lines showed decreased DNA methylation primarily at CpG sites in genic regions, as well as repeated sequences in heterochromatic regions. In addition, transcriptional silencing was released in these plants at most heterochromatin regions examined. Interestingly, the *vim1 vim3* mutant and *vim* knock-down lines gained ectopic CpHpH methylation in the 5S rRNA genes against a background of CpG hypomethylation. The *vim1 vim2 vim3* triple mutant displayed abnormal morphological phenotypes including late flowering, which is associated with DNA hypomethylation of the 5′ region of *FWA* and release of *FWA* gene silencing. Our findings demonstrate that VIM1, VIM2, and VIM3 have overlapping functions in maintenance of global CpG methylation and epigenetic transcriptional silencing.

## Author Summary

Methylation of cytosine bases provides one layer of epigenetic information that is superimposed on the nucleotide sequence of a genome. Proteins that bind methylated cytosines and also help maintain that DNA modification are important linchpins in a self-propagating system mediating memory of epigenetic states. We previously demonstrated that the VIM1 (VARIANT IN METHYLATION 1) protein from the flowering plant *Arabidopsis thaliana* binds DNA that contains methylated cytosine and is required for complete methylation and compaction of centromeric DNA. In this study, we show that VIM1 works in concert with two related proteins, VIM2 and VIM3, to maintain cytosine methylation not only at centromeres but throughout the genome. VIM proteins act specifically in the DNA methylation pathway that targets CpG dinucleotides, which plants share with animals, rather than the plant-specific non-CpG methylation pathways. Loss of *VIM1*, *VIM2*, and *VIM3* function also causes a reduction in transcriptional gene silencing at a variety of sequences, and leads to abnormal developmental phenotypes, including late flowering associated with loss of *FWA* gene silencing. Our results demonstrate that these three related VIM family proteins have overlapping functions in the MET1-mediated CpG methylation pathway.

## Introduction

DNA cytosine methylation is an epigenetic mark important for many processes including parental imprinting, X chromosome inactivation, and the silencing of transposable elements [1]–[3]. In mammals, methylated cytosines are found almost exclusively in symmetrical CpG sequence contexts, and CpG methylation patterns are propagated after DNA replication by “maintenance” DNA methyltransferases (DNMT1-type) [2]. However, DNA cytosine methylation in plants can occur at CpHpG and CpHpH (where H = A, T, or C) as well as CpG sites. In Arabidopsis, these three categories of cytosine methylation are carried out by distinct activities [4]: CpG methylation is maintained primarily by the DNMT1-type methyltransferase, MET1; CHROMOMETHYLASE3 (CMT3) is responsible for CpHpG methylation; and methylation at CpHpH sites is accomplished by a *de novo* methyltransferase, DOMAINS REARRANGED METHYLASE (DRM). Cytosine methylation patterns in Arabidopsis are also trimmed by the action of DNA demethylases in the DEMETER (DME) family, which include REPRESSOR OF SILENCING 1 (ROS1) [5],[6].

Complex interactions between DNA methylation and histone modification articulate epigenetic gene expression states, and methylcytosine-binding proteins play an important role in interpreting the epigenetic information encoded by DNA methylation [3]. The best understood class of methylcytosine-binding proteins contains a conserved methylcytosine-binding domain (MBD) [7]. Several MBD proteins in mammals, including methyl-CpG-binding protein 1 (MeCP1), MeCP2, MBD2, and MBD4 have been identified with high affinity for methylated DNA, and the biological importance of mammalian methylcytosine-binding proteins has been shown in the wide range of severe phenotypes by mutations of these genes [8]–[10]. In contrast to mammalian MBD proteins, knowledge of plant MBD proteins remains relatively limited. Among 13 MBD proteins in the Arabidopsis proteome, three (AtMBD5, AtMBD6, and AtMBD7) have been shown to bind symmetrically methylated CpG sites *in vitro* [11]–[13]. Although developmental defects have been observed in lines carrying a loss-of-function mutation of *AtMBD9* or a transgene directing RNAi against *AtMBD11* transcripts, the role of any AtMBD proteins in epigenetic regulation remains to be defined [14],[15].

Recently a novel class of methylcytosine-binding proteins have been defined that interact with the modified base through an SRA (SET- and RING-Associated) domain [16],[17]. We previously reported that mutations in the Arabidopsis *VIM1* gene, which encodes an SRA domain methylcytosine-binding protein, causes DNA hypomethylation and decondensation of centromeres in interphase [18]. The SRA domain of VIM1 shares amino acid similarity with mammalian UHRF1 (also known as mouse Np95 and human ICBP90), which has been implicated in regulation of chromatin modification [19],[20], transcription [21], and the cell cycle [22]. Recent reports demonstrate that UHRF1 is required for maintenance of CpG DNA methylation [23],[24]. UHRF1 physically interacts with DNMT1 and has been postulated to mediate the loading of DNMT1 on to replicating heterochromatin [23]–[25].

In the Arabidopsis genome, there are four genes that share high sequence similarity with VIM1. To obtain further insight into the function and potential redundancy among the VIM proteins, we have investigated *vim* double and triple mutants, as well as transgenic *vim* knock-down lines that down-regulate multiple *VIM* family genes. Our results indicate that VIM1, VIM2 and VIM3 have overlapping functions in maintenance of cytosine methylation at CpG dinucleotides distributed throughout the genome. Moreover, VIM proteins are required for transcriptional silencing of a variety of sequences, including centromeric repeats, transposons, and the parentally imprinted *FWA* locus.

## Results

### The *VIM* Gene Family in Arabidopsis

*VIM1* is a member of a small gene family, which was originally identified by a naturally-occurring null mutation in the *Arabidopsis thaliana* accession, Borky-4 (Bor-4) [18]. The Arabidopsis genome contains five *VIM* genes, each of which encodes a protein containing a PHD domain, two RING domains, and an SRA domain (Figure S1A). Interestingly, four of the *VIM* genes (*VIM1* [*At1g57820*], *VIM2* [*At1g66050*], *VIM4* [*At1g66040*], and *VIM5* [*At1g57800*]) are located within a 3 Mb region on the lower arm of chromosome 1. A reverse transcriptase pseudogene (*At1g57810*) is located between *VIM1* and *VIM5*, and an unrelated putative pseudogene (*At1g66045*) is located between *VIM2* and *VIM4*. The VIM proteins share high amino acid sequence identity (>68%) throughout their entire length, including the four previously recognized domains (Figure S1A).

We measured the steady state levels of transcripts using reverse transcriptase (RT)-PCR from all five *VIM* genes to obtain insight into their function and potential redundancy (Figure S1B and S1C). *VIM1* was highly expressed in inflorescence tissue and to a lesser extent in two-week-old leaves of wild-type Columbia (Col) plants (Figure S1C). *VIM3* [*At5g39550*] transcripts were found in both inflorescences and two-week-old leaves, while *VIM2* was abundantly expressed in inflorescences. In contrast, *VIM4* and *VIM5* were absent from leaves or inflorescence tissue at this level of detection, suggesting that the steady-state levels of *VIM4* and *VIM5* are very low or that these may be pseudogenes. Based on our expression data, we concentrated on the functional analysis of *VIM1*, *VIM2*, and *VIM3*.

### VIM1, VIM2, and VIM3 Function Redundantly to Maintain Centromere DNA Methylation

**Figure 1:**
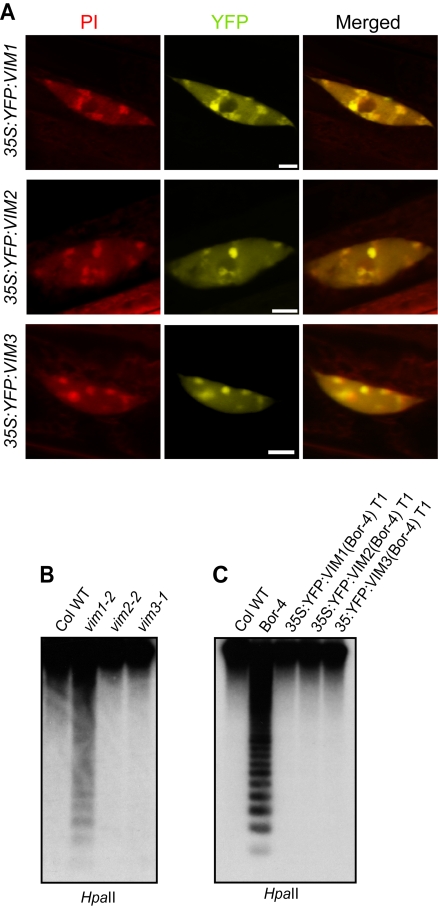
DNA methylation of the centromeric repeats in *vim* mutants. (A) Subnuclear localization of VIM proteins. Localization of VIM proteins were detected in Col root nuclei expressing a YFP-VIM1, YFP-VIM2, or YFP-VIM3 transgene under the control of the 35S promoter. Propidium iodide (PI) was used as a DNA counterstain; chromocenters are more intensely stained. Bar, 5 µm. (B) 180-bp centromeric repeat methylation phenotype in different *vim* single mutants. (C) Suppression of centromere DNA hypomethylation phenotype in the Bor-4 strain, which contains the natural *vim1-1* allele, by overexpressing *VIM* cDNA clones. Bor-4 plants were transformed with *VIM1*, *VIM2*, or *VIM3* transgene under the control of the 35S promoter. Genomic DNA samples of the indicated genotypes were digested with *Hpa*II and used for DNA gel blot analysis with a probe for the 180-bp centromere repeats.

We first investigated the subnuclear localization of VIM1, VIM2, and VIM3 in interphase. Trangenes containing *VIM1*, *VIM2*, or *VIM3* cDNAs fused with *YFP* at the N-terminus under the control of the strong, constitutive cauliflower mosaic virus (CaMV) 35S promoter were transformed into Col plants. Fixed nuclei from transgenic Col root cells expressing YFP-VIM1, YFP-VIM2, or YFP-VIM3 are shown in Figure 1A. YFP-VIM1, -VIM2, and -VIM3 fusion proteins were broadly distributed in the nucleus and were enriched in the heterochromatic chromocenters. The similar subnuclear localization of all three expressed VIM proteins suggests a possible functional redundancy among these VIM proteins.

We characterized T-DNA insertional mutations that disrupt the coding sequences of *VIM1*, *VIM2*, and *VIM3* in the Col background. Using RT-PCR and primers flanking the T-DNA insertion sites, we did not detect expression of *VIM1*, *VIM2*, and *VIM3* in *vim1-2*, *vim2-2*, and *vim3-1* mutant plants, respectively, confirming that the T-DNA insertions likely destroy gene function (Figure S2 and data not shown). While the *vim1-2* mutation caused a slight decrease in DNA methylation of centromeric 180-bp repeat arrays, neither the *vim2-2* nor the *vim3-1* allele led to a centromere repeat hypomethylation phenotype (Figure 1B). Therefore, VIM2 and/or VIM3 are not required for centromere DNA methylation, or alternatively, one or both of these genes function redundantly with VIM1 to maintain centromere DNA methylation.

To test these alternative hypotheses, we individually introduced cDNA copies of Col *VIM1*, *VIM2* or *VIM3* genes into Bor-4 plants, which carry the *vim1-1* loss-of-function allele, and investigated the effect on centromere DNA methylation. *VIM* cDNAs fused with *YFP* at the N-terminus were expressed under the control of the 35S promoter (Figure S3). Expression of a wild-type Col *VIM1* cDNA in Bor-4 plants fully restored DNA methylation at the centromere (Figure 1C). Over-expression of Col *VIM2* or *VIM3* cDNAs can also fully suppress the hypomethylation of centromeric repeats in Bor-4 *vim1-1* plants, demonstrating that VIM2 and VIM3 proteins can function redundantly with VIM1.

### Isolation of *vim* Double Mutants and Transgenic Lines with Coordinate Gene Silencing of *VIM1*, *VIM2*, and *VIM3*

To determine the extent of functional redundancy within the *VIM* gene family, we generated and characterized a set of double mutants combining two loss-of-function mutations among *VIM1*, *VIM2*, and *VIM3*. As an alternative approach, we also isolated transgenic plants with a coordinate decrease in the expression of *VIM1*, *VIM2* and *VIM3* genes. We took advantage of the variation in transgene expression among the Col transgenic lines expressing an *YFP-VIM1* cDNA used in our nuclear localization analysis (Figure 1A). A significant proportion of the transgenic plants did not show any YFP expression and had lower steady-state transcript levels of *VIM2* and *VIM3* as well as *VIM1* (Figure S2) presumably due to RNA interference. We chose two of these transgenic *VIM* family “knock-down” lines (*vim*KD-A and *vim*KD-B) for further analysis.

### DNA Methylation in Heterochromatic Regions Is Decreased in the *vim1 vim3* Double Mutant and Transgenic *vim* Knock-Down Lines

**Figure 2:**
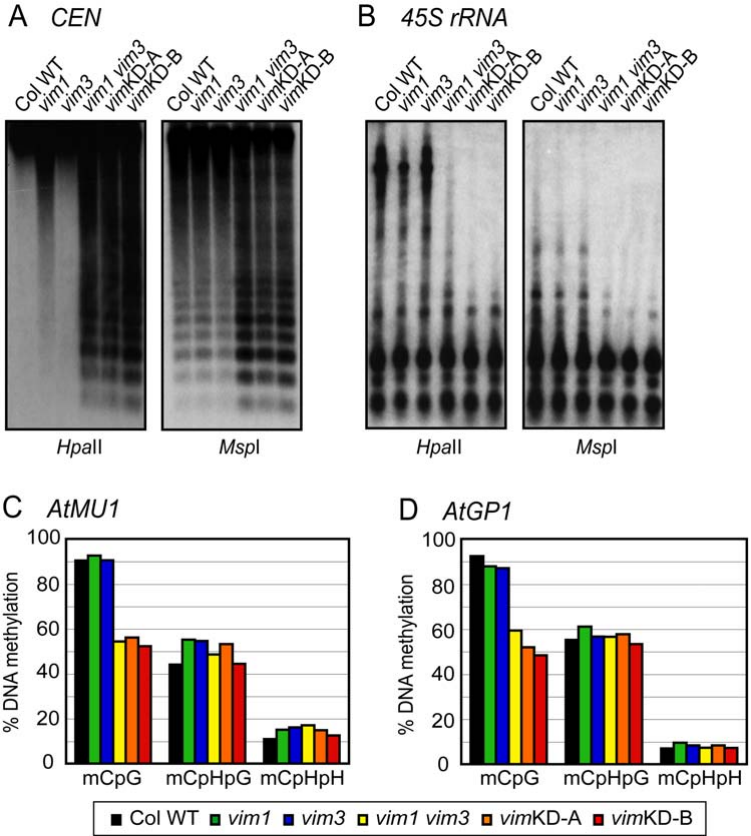
DNA methylation at heterochromatic loci is affected in the *vim1 vim3* mutant and the *vim* knock-down lines. (A, B) DNA methylation was determined by DNA gel blot analysis; genomic DNA was digested with *Hpa*II or *Msp*I and blots were hybridized with probes corresponding to 180-bp centromeric repeats (A) and 45S rRNA (B). (C, D) DNA methylation for *AtMU1* (C) and *AtGP1* (D) was analyzed by bisulfite sequencing. Histograms represent the percentage of CpG, CpHpG, and CpHpH methylation in the indicated genotypes.

We tested whether DNA methylation at highly repetitive loci is dependent on the function of multiple *VIM* gene family members. Genomic DNA samples prepared from plants of the different *vim* genotypes were analyzed by DNA gel blot hybridization after digestion with methylation-sensitive restriction endonucleases. Cleavage by *Hpa*II (5′-CCGG-3′) is inhibited by either CpG or CpHpG methylation; *Msp*I (5′-CCGG-3′) digestion is blocked by CpHpG methylation; and *Nla*III (5′-CATG-3′) activity is inhibited by CpHpH methylation. The *vim1 vim2* and *vim2 vim3* double mutants did not display any change in centromere or 45S rRNA gene cytosine methylation compared to *vim1* and Col wild-type plants, respectively (Figure S4 and data not shown). In contrast, the *vim1 vim3* double mutant and the two *vim* knock-down lines displayed decreased CpG and CpHpG methylation at *Hpa*II and *Msp*I sites in both the centromere and 45S rRNA genes relative to the *vim1* single mutant (Figure 2A and 2B). There was no difference among the genotypes tested after *Nla*III digestion at the 180-bp centromeric repeats and 45S rRNA genes (Figure S5), which might reflect a low level of CpHpH methylation. Our DNA blot data demonstrate that VIM proteins, particularly VIM1 and VIM3, function redundantly to maintain CpG and CpHpG methylation at the 180-bp centromere repeats, as well as in repetitive sequences outside the centromere.

To evaluate the potential role of the *VIM* genes in shaping DNA methylation patterns in other heterochromatic regions, the DNA methylation status of two transposable elements, the MULE DNA transposon *AtMU1* and the *gypsy*-class LTR retroelement *AtGP1*, was compared among the different *vim* genotypes using bisulfite sequencing. We found that CpG sites in these elements were heavily methylated in Col wild-type, *vim1*, and *vim3* plants, but CpG methylation was significantly decreased in the *vim1 vim3* mutant and the two transgenic *vim* knock-down lines (Figure 2C and 2D; Table S1). In contrast, no substantial changes in CpHpG and CpHpH methylation were observed. These results further indicate that VIM proteins function redundantly to maintain cytosine methylation of heterochromatic sequences outside of the centromere.

### 5S rRNA Gene Methylation Is Altered in the *vim1 vim3* Mutant and Transgenic *vim* Knock-Down Lines

**Figure 3:**
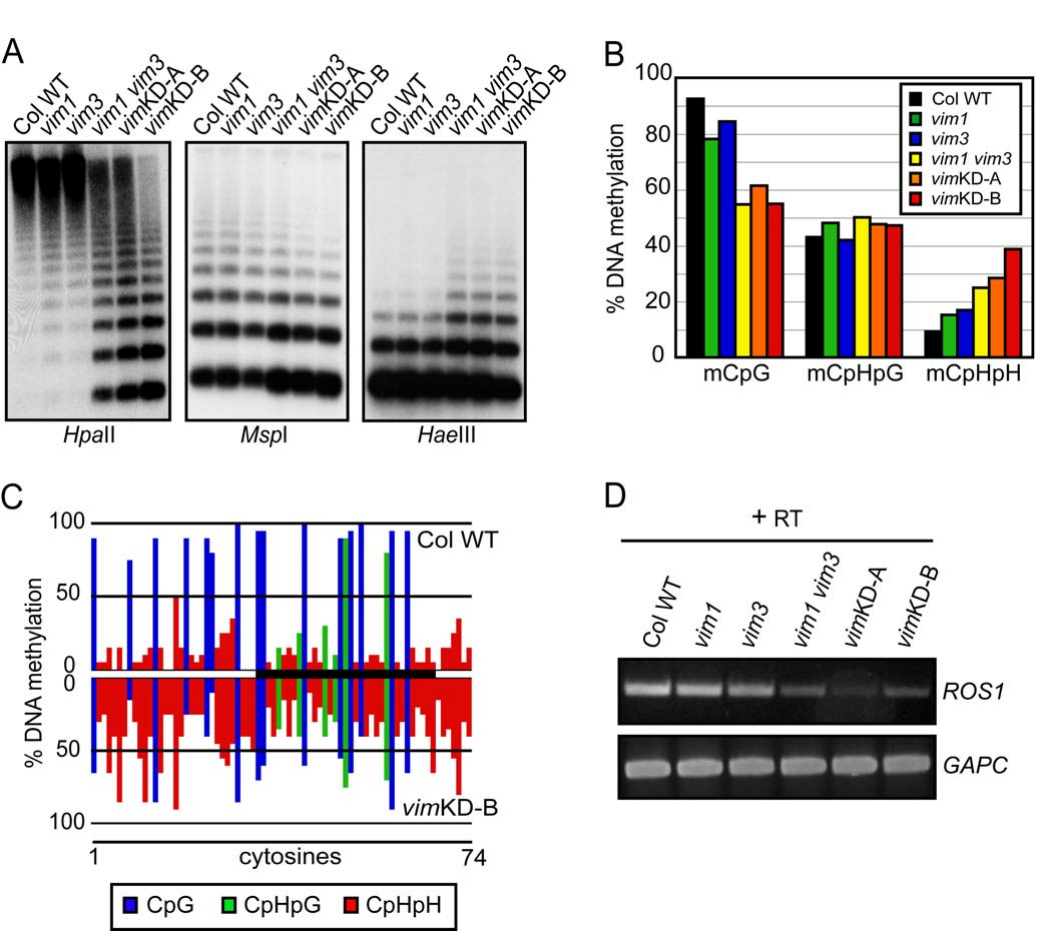
DNA methylation patterns at 5S rRNA genes. (A) DNA methylation was determined by DNA gel blot analysis with genomic DNA samples; genomic DNA digested with *Hpa*II, *Msp*I, or *Hae*III was hybridized to a 5S rRNA probe. (B) Analysis of DNA methylation by bisulfite sequencing. Histograms represent the percentage of CpG, CpHpG, and CpHpH methylation in the indicated genotypes. (C) Detailed DNA methylation profile for 5S rRNA gene in Col (top) and *vim*KD-B (bottom). The percentages of methylation of each cytosine residue were calculated. The x-axis represents cytosine positions in the analyzed region, and the y-axis represents methylation levels in each genotype. The black bar in the middle shows a coding region of 5S rRNA gene. CpG, CpHpG, and CpHpH methylation are indicated by blue, green, and red bars, respectively. (D) *ROS1* mRNA accumulation was determined by RT-PCR. Total RNA from 3-week-old leaves of the indicated plant genotypes, and then RT-PCR was carried. First-strand cDNA synthesis was carried out with or without RT; we did not detect transcripts from the ‘minus RT’ samples. Amplification of *GAPC* (glyceraldehyde-3-phosphate dehydrogenase) was used to normalize RNA template amounts.

Next, we assessed the DNA methylation pattern of 5S rRNA genes using DNA gel blot hybridization analysis and bisulfite sequencing. The *vim1 vim3* mutant and the two transgenic *vim* knock-down lines had strongly decreased CpG methylation at *Hpa*II sites and reduced CpHpG methylation at *Msp*I sites in the 5S rRNA genes (Figure 3A). This hypomethylation was accompanied by CpHpH hypermethylation evidenced by higher molecular weight hybridization signals after *Hae*III (5′-GGCC-3′) digestion (Figure 3A) and *Nla*III digestion (Figure S5). Bisulfite sequencing of the 5S rRNA genes confirmed that CpG methylation was significantly decreased in the *vim1 vim3* mutant and the two transgenic *vim* knock-down lines relative to Col wild-type plants (Figure 3B; Table S1). Most of the CpG sites are affected by the *vim1 vim3* mutation combination and the coordinate knock-down of *VIM* gene expression in the transgenic lines, but the degree of hypomethylation at different CpG sites varied widely (5%–70% decrease in *vim*KD-B) (Figure 3C and data not shown). Our bisulfite sequencing analysis also demonstrated an increase in CpHpH methylation throughout the 5S rRNA genes in the *vim1 vim3* mutant and the transgenic *vim* knock-down lines, especially *vim*KD-B.

Increased CpHpH methylation levels observed in the 5S rRNA genes could result from activation of DNA methylation activity and/or inhibition of DNA demethylation activity. To address these possibilities, we first monitored the levels of siRNA species that could target *de novo* methylation [26] to the 5S rRNA repeats, but found no significant changes in the abundance of siRNA from 5S rRNA (Figure S6). We also examined the steady-state levels of transcripts from the three major DNA methyltransferase genes (*CMT3*, *DRM2*, and *MET1*) and two DNA demethylase genes (*DME* and *ROS1*) by RT-PCR. Although no significant changes in steady-state transcript levels for the three DNA methyltransferase genes or *DME* were observed in any *vim* mutant (data not shown), *ROS1* transcript accumulation was reduced in the *vim1 vim3* mutant and the transgenic *vim* knock-down lines (Figure 3D). This result raises the possibility that *ROS1* transcriptional repression in plants deficient in activity of the VIM proteins might contribute to CpHpH DNA hypermethylation in the 5S rRNA genes.

### VIM Proteins Affect Genic DNA Methylation at CpG Sites

**Figure 4:**
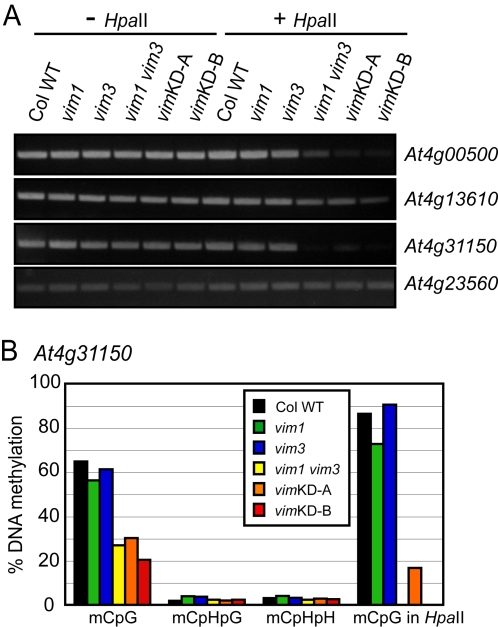
Genic DNA methylation is decreased only at CpG in the *vim1 vim3* mutant and the *vim* knockdown lines. (A) DNA methylation analysis by *Hpa*II-PCR. *Hpa*II-digested genomic DNA was amplified by PCR with primers for the indicated genes. Undigested DNA (- *Hpa*II) and a gene lacking *Hpa*II sites (*At4g23560*) served as PCR controls. (B) Analysis of DNA methylation at *At4g31150* by bisulfite sequencing. Histograms represent the percentage of methylation of CpG, CpHpG, CpHpH, and CpG methylation at the *Hpa*II site tested in (A).

We analyzed genic cytosine methylation at three different loci [27], *At4g00500* (lipase class 3 family protein), *At4g13610* (MEE57, maternal effect embryo arrest 57), and *At4g31150* (endonuclease V family protein), to determine whether VIM proteins are involved in DNA methylation of low-copy, expressed sequences not associated with heterochromatin. First, a PCR-based method was used to assay DNA methylation of these loci in the *vim* mutant lines. After *Hpa*II treatment, unmethylated DNA will be digested and therefore not amplified by PCR. In wild-type Col, *vim1*, and *vim3* samples, all three tested genic regions were easily amplified after *Hpa*II digestion. In contrast, the abundance of PCR product for *At4g00500* and *At4g31150* (and to a lesser extent *At4g13610*) amplified from the *vim1 vim3* and transgenic *vim* knock-down samples was lower than that from the wild-type Col sample, indicating a reduction in CpG methylation (Figure 4A).

To verify and extend the results obtained by the PCR-based method, bisulfite sequencing was carried out for the *At4g31150* gene (Figure 4B; Table S1). DNA methylation at *At4g31150* in wild-type Col plants was predominantly localized in CpG dinucleotides. The *vim1 vim3* mutant and the transgenic *vim* knock-down lines showed a two- to three-fold reduction in CpG methylation at the locus. In wild-type Col, *vim1*, and *vim3* samples, 73–91% of the CpG dinucleotide in the *Hpa*II site of *At4g31150* monitored in our PCR-based assay was methylated. Notably, less than 17% of those sites were methylated in *vim*KD-A, and the CpG in the *Hpa*II site was completely unmethylated in the *vim1 vim3* mutant and the *vim*KD-B line. In contrast to CpG methylation, wild-type Col samples had very low levels (<4%) of CpHpG and CpHpH methylation at *At4g31150*, and none of the tested genotypes displayed an effect on CpHpG and CpHpH methylation. These data confirm that VIM deficiency primarily affects CpG methylation, and demonstrate that the targets of VIM activity are broadly distributed in the genome to include genic regions.

### Transcriptional Silencing Is Released in the *vim1 vim3* Mutant and Transgenic *vim* Knock-Down Lines

**Figure 5:**
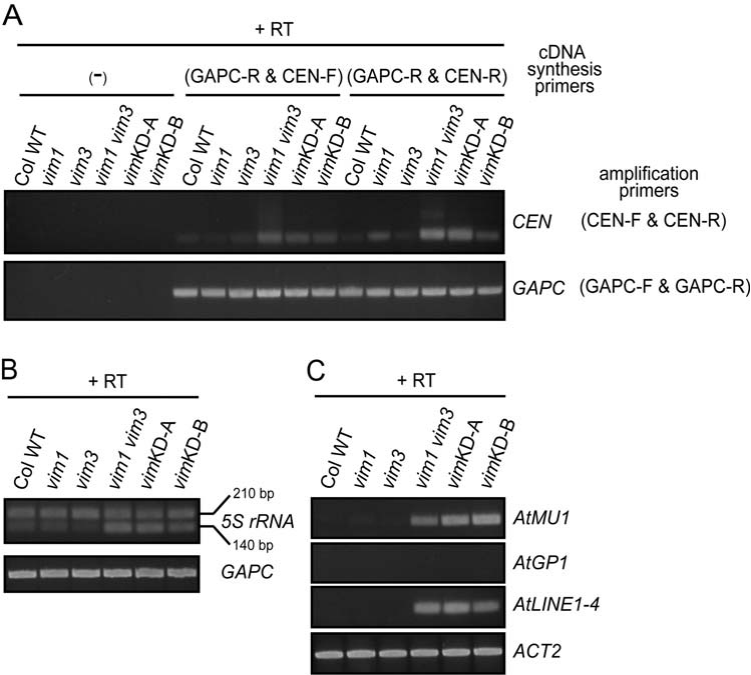
Release of transcriptional silencing at heterochromatic regions in the *vim1 vim3* mutant and the *vim* knockdown lines. (A) Transcripts from the 180-bp centromeric repeats. RT was performed with the primers indicated to the top of the panel (cDNA synthesis primers), followed by PCR using the amplification primers. *GAPC* was used as a control. (-), no primers; GAPC-R, GAPC reverse primer; CEN-F, centromere forward primer; CEN-R, centromere reverse primer. (B) RT-PCR detection of 5S-210 and 5S-140 transcript levels. RT was performed with GAPC-R and 5S rRNA reverse primer with or without RT followed by PCR with the first primer and a corresponding primer on the other strand. First-strand cDNA synthesis was carried out with or without RT; we did not detect transcripts from the ‘minus RT’ samples. (C) Transcription analysis of various transposons. RT-PCR was carried out using RNA samples from the indicated genotypes. First-strand cDNA synthesis was carried out with or without RT; we did not detect transcripts from the ‘minus RT’ samples. Amplification of *ACT2 (Actin2)* was used to normalize RNA template amounts.

RT-PCR was carried out to determine whether VIM deficiency affects gene expression. First, we investigated the level of transcripts generated from the 180-bp centromeric repeats in different *vim* genotypes. Elevated levels of 180-bp repeat transcripts from both strands were detected in the *vim1 vim3* mutant and the two transgenic *vim* knock-down lines, suggesting that DNA hypomethylation of the 180-bp centromeric repeats caused by VIM deficiency is associated with a loss of transcriptional silencing of these repeats (Figure 5A). Next, we focused on the 5S rRNA genes, where loss of VIM function leads to dramatic changes in cytosine methylation. 5S rRNA genes are organized into pericentromeric tandem repeat arrays, and only a subset of the repeats is transcribed with the remainder being epigenetically silenced. To detect the release of 5S rRNA repeat silencing, we used primer pairs that detect two 5S transcript variants, resulting in amplification of 140 and 210 nucleotide products from cDNA templates prepared from wild-type Col plants. 5S-140 transcripts accumulated to a higher level in the *vim1 vim3* mutant and the transgenic *vim* knock-down lines compared to the wild-type Col genotype, but no significant increase in 5S-210 was observed (Figure 5B). This result demonstrates that a decrease of 5S rRNA gene methylation at CpG sites in the *vim1 vim3* mutant and the transgenic *vim* knock-down lines is associated with loss of 5S rRNA gene silencing of the smaller variant. We also examined the expression of the two loci representing methylated genes: *At4g00500* and *At4g31150*. We detected equivalent levels of transcription for *At4g00500* and *At4g31150* in each genotype (Figure S7). These data suggest that the genic CpG methylation found in these genes does not lead to a general repression of gene expression and that VIM proteins are not playing an active role in regulating expression of these genes.

We also examined dispersed transposable elements for loss of transcriptional silencing. Specifically, we investigated the transcription status of the non-LTR retroelement *AtLINE1-4*, the LTR retrotransposon *AtGP1*, and the class II element *AtMU1* using an RT-PCR assay on RNA samples derived from *vim* mutants (Figure 5C). No *AtGP1* transcripts were detected in any of the *vim* mutant lines despite the loss of CpG methylation. However, we found that transcription of *AtMU1* and *AtLINE1-4* elements were activated in the *vim1 vim3* mutant and the transgenic *vim* knock-down lines. These findings indicate that VIM proteins function redundantly to silence different types of transposable elements.

### The Late Flowering Phenotype of the *vim1 vim2 vim3* Triple Mutant Is Associated with Ectopic *FWA* Expression

**Figure 6:**
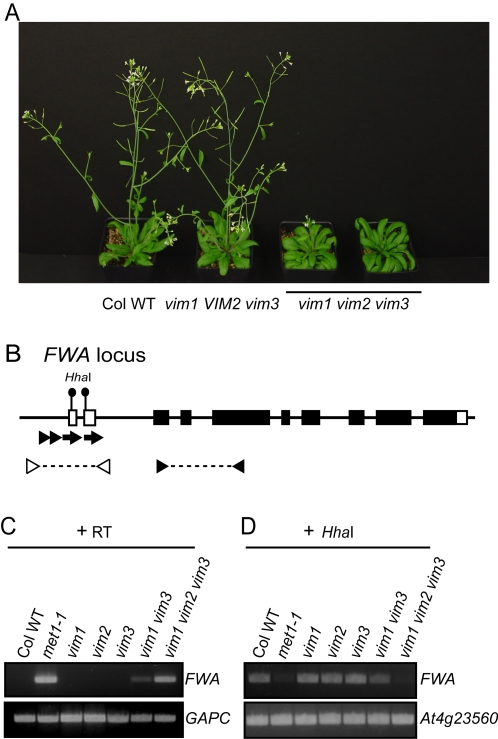
Loss of *FWA* silencing in the *vim1 vim2 vim3* triple mutant. (A) Delayed flowering of *vim1 vim2 vim3* mutant plants. A *vim1/vim1 vim2/+vim3/vim3* parent was self-pollinated to generate a segregating family and triple mutants were compared to *vim1 VIM2 vim3* siblings (*VIM2* stands for +/+ or *vim2*/+), which were indistinguishable from wild-type Col plants (Col WT) with respect to flowering time and overall morphology. Image of 30-day old plants grown under long-day conditions. (B) *FWA* gene structure. Solid rectangles, translated exons; open rectangles, untranslated exons; arrowheads and arrows, direct repeats; triangles, primers used to amplify the 5′ region of *FWA* or *FWA* coding sequences. (C) Ectopic expression of *FWA* in rosette leaves of the *met1-1*, *vim1 vim3*, and *vim1 vim2 vim3* mutants. *FWA* transcripts were examined by RT-PCR and the constitutively expressed *GAPC* served as a control. First-strand cDNA synthesis was carried out with or without RT; we did not detect transcripts from the ‘minus RT’ samples. (D) DNA methylation of the *FWA* upstream region. Genomic DNA was digested with *Hha*I; subsequently, the tandem repeats in the 5′ region of *FWA* were amplified by PCR. A gene lacking *Hha*I sites (*At4g23560*) served as a PCR control.

Despite the changes in DNA methylation and the release of gene silencing, the *vim1* single mutant, the *vim1 vim3* double mutant, and the transgenic *vim* knock-down lines did not display abnormal developmental phenotypes under standard growth conditions (data not shown). The lack of morphological phenotypes may be due to incomplete ablation of the expressed VIM family genes. We tested this hypothesis by combining *vim1*, *vim2*, and *vim3* mutations in a single background and found that the triple mutant has distinct morphological phenotypes, including late flowering (Figure 6A and Figure S8) and production of aerial rosettes on the flowering stem (data not shown). We investigated the basis of the late flowering phenotype by examining the imprinted locus *FWA*, which is demethylated and reactivated in DNA hypomethylation mutants (*e.g.*, *met1*) leading to delayed flowering [28],[29]. As shown in Figure 6 (panels B, C and D), homozygotes carrying the hypomorphic *met1-1* allele show ectopic *FWA* expression in vegetative tissues and hypomethylation of the transposon-related repeat sequences that comprise the promoter region. A low level of ectopic *FWA* expression, apparently insufficient to affect flowering, was observed in the *vim1 vim3* mutant sample, correlated with partial hypomethylation of the upstream region of the *FWA* gene (Figure 6C and 6D). The more extensive hypomethylation of the *FWA* upstream region in the *vim1 vim2 vim3* triple mutant was associated with stronger *FWA* expression, which is expected to cause late flowering.

### The *vim1 vim2 vim3* Triple Mutant Displays DNA Hypomethylation Phenotypes Comparable to Those Exhibited by a *met1* Null Mutant

**Figure 7:**
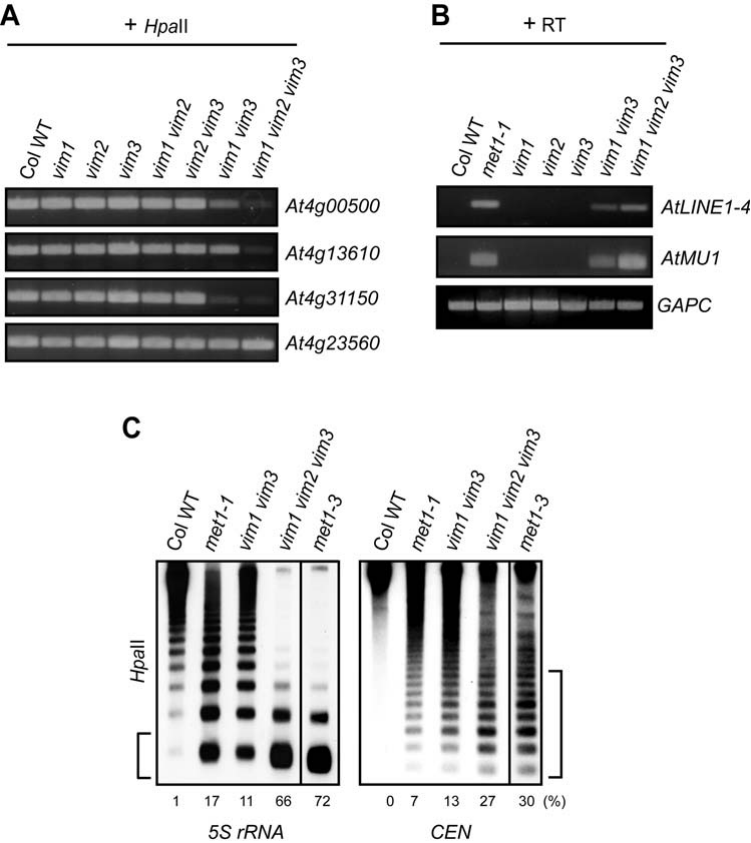
DNA hypomethylation and transcriptional reactivation in the *vim1 vim2 vim3* mutant. (A) Genic DNA methylation in *vim* mutants. DNA methylation was analyzed by *Hpa*II-PCR. *Hpa*II-digested genomic DNA was amplified by PCR with primers for the indicated genes. A gene lacking *Hpa*II sites (*At4g23560*) served as PCR controls. (B) Release of transcriptional silencing of transposons. RT-PCR was carried out using RNA samples from the indicated genotypes. First-strand cDNA synthesis was carried out with or without RT; we did not detect transcripts from the ‘minus RT’ samples. Amplification of *GAPC* was used to normalize RNA template amounts. (C) DNA methylation in 5S rRNA genes and the 180-bp centromeric repeats (*CEN*) in *vim* and *met1* mutants. DNA methylation was monitored by DNA gel blot analysis; genomic DNA was digested with *Hpa*II and the blot was hybridized sequentially with radiolabeled probes corresponding to 5S rRNA (left) and the 180-bp centromeric repeats (right). More genomic DNA for the *met1-3* mutant was loaded on the gel, so a matched exposure is shown for this sample as a separate box. The numbers shown below the each panel indicate the percentage of hybridization signal present in the bottom band for 5S rRNA genes (denoted by bracket on the left) and in the lower 8 bands for the 180-bp centromere repeats (denoted by bracket on the right).

As seen in Figure 6, the *vim1 vim2 vim3* triple mutant showed a stronger phenotype than that observed in the *vim1 vim3* double mutant, indicating VIM2 is important in repressing *FWA* expression and hypermethylation of the upstream region of the *FWA* gene in vegetative tissues. To investigate whether VIM2 has a redundant function with VIM1 and VIM3 outside of the *FWA* locus, we examined DNA methylation in other genic regions. The abundance of PCR product for *At4g00500* and *At4g13610* amplified from the *Hpa*II-digested *vim1 vim2 vim3* mutant sample was significantly less than that from the *vim1 vim3* mutant sample, indicating that the *vim* triple mutant displayed a more severe reduction in genic methylation compared to the *vim1 vim3* mutant (Figure 7A).

As the *vim* triple mutant phenocopied *met1* mutants in several aspects, including preference for CpG hypomethylation, ectopic CpHpH hypermethylation, reduced *ROS1* expression, and late flowering associated with ectopic expression of *FWA*, we further explored the severity of phenotypes displayed by *vim* mutants relative to *met1* mutants. Specifically, we compared transcriptional activation of transposons and DNA hypomethylation of highly repetitive heterochromatic regions in *vim1 vim2 vim3* and *met1* mutants. First, we re-examined the transcription status of *AtLINE1-4* and *AtMU1* using an RT-PCR assay. Loss of transcriptional silencing of *AtMU1* and *AtLINE1-4* elements was more severe in the *vim1 vim2 vim3* mutant compared to the *vim1 vim3* mutant (Figure 7B). Transcription of both transposons in the *vim1 vim2 vim3* mutant was similar or higher than that observed in the hypomorphic *met1-1* mutant. We next assessed the DNA methylation pattern of the 180-bp centromere repeats and the 5S rRNA genes by DNA gel blot hybridization analysis after digestion with *Hpa*II. The *vim1 vim2 vim3* triple mutant displayed stronger hypomethylation at *Hpa*II sites relative to the *vim1 vim3* double mutant for both repeat families (Figure 7C). Interestingly, the DNA hypomethylation phenotypes in the *vim1 vim2 vim3* triple mutant were significantly stronger than those observed in the hypomorphic *met1-1* mutant, but similar to the phenotypes exhibited by the *met1-3* null mutant. Taken together, these findings indicate that VIM1, VIM2 and VIM3 function redundantly to silence different types of transposable elements and to maintain CpG methylation in the heterochromatic as well as genic regions. Further, our results indicate that simultaneous loss of *VIM1*, *VIM2* and *VIM3* function almost completely blocks CpG methylation.

## Discussion

The Arabidopsis genome contains five *VIM* genes, each of which encodes a protein containing a PHD domain, two RING domains, and an SRA domain. Previously, we reported that VIM1 is an unconventional methylcytosine-binding protein and that loss-of-function *vim1* mutations cause cytosine hypomethylation and decondensation of centromeres [18]. Here we describe a broader analysis of the *VIM* gene family in Arabidopsis. Our results indicate that the expressed VIM proteins cooperate to maintain global CpG methylation and epigenetic transcriptional silencing.

### Functional Redundancy in the *VIM* Gene Family

We previously reported that VIM1 binds methylated CpG and CpHpG *in vitro* and similarly Johnson *et al.* [16] showed that ORTH1/VIM3 and ORTH2/VIM1 can bind methylated CpG, CpHpG, or CpHpH substrates. Our results provide additional support for the hypothesis that VIM1, VIM2, and VIM3 function redundantly. First, all three expressed VIM proteins showed a similar subnuclear localization. When ectopically expressed in transgenic plants under control of the 35S promoter, YFP-VIM1, VIM2, and VIM3 protein fusions were broadly distributed in the nucleus and were enriched in the heterochromatic chromocenters (Figure 1A). Second, *VIM1*, *VIM2*, and *VIM3* were abundantly expressed in leaves and inflorescence tissue – an overlapping expression pattern that supports the possibility of functional redundancy (Figure S1C). Third, the heterologous overexpression of wild-type Col *VIM2* or *VIM3* coding sequences compensated for the loss of *VIM1* with regard to centromere DNA methylation when transformed into Bor-4 *vim1-1* plants (Figure 1C). Fourth, combining *vim* mutations or suppressing the expression of multiple *VIM* genes in transgenic lines resulted in stronger DNA hypomethylation than those exhibited by *vim* single mutants (Figure 2, 3, 4, 6, and 7). A release of gene silencing also occurred at the 180-bp centromeric repeats, 5S rRNA repeats, and some transposable elements in the *vim1 vim3* double mutants and the transgenic *vim* knock-down lines (Figure 5). Simultaneous disruption of *VIM1*, *VIM2* and *VIM3* led to a loss of *FWA* gene silencing in vegetative tissues, as well as a more severe reduction in genic and tandem repeat methylation compared to the *vim1 vim3* double mutant (Figure 6 and 7). These results indicate that VIM proteins play important, overlapping roles in maintenance of cytosine methylation and transcriptional silencing throughout the Arabidopsis genome.

Although VIM proteins have overlapping functions, a hierarchy exists among VIM proteins. The *vim1 vim3* mutant displayed a strong synergistic effect on cytosine methylation compared to either single mutant, whereas the *vim1 vim2* mutation combination showed no significant enhancement of DNA hypomethylation compared to the *vim1* single mutant (Figure S4 and Figure 7A). The importance of VIM2 is demonstrated by the more severe cytosine hypomethylation and loss of transcriptional silencing displayed by the *vim1 vim2 vim3* triple mutant compared to the *vim1 vim3* double mutant (Figure 6 and 7). These results indicate that VIM1 is the major functional member of the VIM family with regards to DNA methylation and epigenetic silencing, while VIM3 and VIM2 (in descending order) play lesser roles in the examined loci. This functional hierarchy might reflect qualitative differences among VIM proteins or relative gene expression levels (*VIM1* is the most highly transcribed member of the gene family based on public databases: http://mpss.udel.edu/at and http://bbc.botany.utoronto.ca/efp).

### Specificity of Cytosine Hypomethylation in *vim* Mutants

VIM proteins affect CpHpG as well as CpG methlyation at the 180-bp repeats and 45S rRNA genes (Figure 2), while VIM proteins specifically affect CpG methlyation in other loci examined. One possibility is that CpHpG methylation in the centromere and 45S rRNA genes may be reduced as a secondary consequence of a loss of CpG methylation, as reported in *met1* mutants [29]. Alternatively, VIM proteins may have locus-specific regulatory mechanisms for maintaining DNA methylation. One indication of locus specificity is the preferential effect of *vim1* mutations on centromere methylation and compaction [18], which might result from varying levels of functional redundancy at different genomic locations – for instance, a more diminished role for VIM2 and VIM3 at the centromere. The specificity may be determined by the primary sequence itself or sequence copy number. In addition, the activities of VIM proteins could be influenced by other proteins that have sequence specificity.

### VIM and Flowering Time Control

The *vim1 vim2 vim3* triple mutant showed delayed flowering, and we uncovered *FWA* hypomethylation and ectopic *FWA* expression as one possible mechanism for this developmental phenotype. Liu *et al.* reported that plants overexpressing a *VIM1*-GFP fusion protein leads to delayed flowering and an elevated level of a key repressor of flowering, *FLOWERING LOCUS C* (*FLC*) transcripts [30]. One possibility is that the late flowering phenotypes in *vim1 vim2 vim3* triple mutant plants and plants overexpressing *VIM1* result from different mechanisms. Another possibility is that the late flowering phenotype in the VIM1 overexpression line might be caused by a dominant negative mechanism. The overexpressed VIM1-GFP fusion proteins could sequester other components for maintaining DNA methylation and epigenetic silencing. Alternatively, the *VIM1* transgene induce silencing of endogenous *VIM* genes, similar to the situation in our *vim*KD-A and *vim*KD-B transgenic lines.

### Possible Mechanisms of VIM Action

The precise role of VIM proteins in epigenetic regulation remains an open question, but two plausible models are supported by previous reports and this study. The mammalian counterparts of VIM proteins exhibit a variety of activities that either directly modify histones [17],[19] or specifically recognize modified histones [31]. Accordingly, we originally proposed that VIM1 affects DNA modification by acting at the chromatin – cytosine methylation interface, possibly by modifying chromatin substrates for DNA methyltransferase activity. The preference for CpG hypomethylation in the *vim* mutants suggests that the MET1-mediated pathway is primarily affected by any alteration of the chromatin substrates. In the alternative model, which is based on the physical interaction of mammalian VIM homologs with DNMT1 [23]–[25], VIM proteins tether the DNMT1-class CpG methyltransferase MET1 to the replication fork. This model predicts that inactivation of the redundant VIM family proteins would phenocopy *met1* mutants – a prediction supported by several observations. First, the *vim1 vim3* mutant and the transgenic *vim* knock-down lines showed a preference for CpG hypomethylation, similar to *met1* mutants. Second, these *vim* mutant lines exhibited CpHpH hypermethylation and a reduction in *ROS1* transcript level, two recently described characteristics of *met1* null mutants [32],[33]. Third, the developmental phenotypes of the *vim1 vim2 vim3* mutant, including late flowering, resembled that of *met1* homozygotes [28],[29]. The late flowering phenotype is likely to be caused in part by the ectopic expression of *FWA* associated with DNA hypomethylation of the upstream repeats observed in both *met1* and *vim1 vim2 vim3* mutants. Fourth, the reduction in 180-bp centromere and 5S rRNA gene repeat methylation in the *vim1 vim2 vim3* triple mutant matched the extreme hypomethylation observed in the *met1-3* null mutant. The parallels between the *vim1 vim2 vim3* mutant and *met1* mutants argue that the VIM proteins are essential components of the MET1-mediated cytosine methylation pathway.

## Materials and Methods

### Plant Materials and Growth Conditions

Seeds of *vim* T-DNA insertion mutants were obtained from the SALK T-DNA collection [34] through the Arabidopsis Biological Resources Center at The Ohio State University. The *vim1-2* allele (SALK_050903), the *vim2-2* allele (SALK_133677), and the *vim3-1* allele (SALK_088570) carry a T-DNA insertion in the fourth exon, the third exon, and the fourth exon of the corresponding gene, respectively. Dr. Jerzy Paszkowski kindly donated the *met1-3* mutant seeds. Plants were grown in a controlled environmental chamber at 22°C under long-day conditions (16 h light per day).

### Construction of Plant Expression Vectors and Generation of Transgenic Plants

Full-length *VIM1*, *VIM2*, and *VIM3* cDNA clones were PCR-amplified from a wild-type Col first-strand cDNA preparation using primers VIM1-F/VIM1-R, VIM2-F/VIM2-R, and VIM3-F/VIM3-R, respectively. The fragments were cloned into pENTR-D TOPO (Invitrogen, USA) and the resulting *VIM* inserts were recombined into pEarlyGate104 [35] using Gateway technology (Invitrogen, USA). These constructs were transformed into *Agrobacterium tumefaciens* (LBA4404) and were introduced into Col WT or Bor-4 *vim1-1* plants by *in planta* transformation [36].

### Localization of YFP-VIM Proteins

T2 generation transgenic seeds were germinated on 1× MS (Murashige and Skoog) media and grown for 6–10 days in 16 h/8 h (light/dark) growth conditions at 22°C. Seedlings were fixed in a 1× PBS solution containing 4% paraformaldehyde for 1 hour at room temperature and subsequently stained with 10 µg/mL propidium iodide. Images of root nuclei were acquired with a Leica SP2 laser scanning confocal microscope equipped with a 488 nm laser, 561 nm laser, and filter sets suitable for the detection of YFP and propidium iodide. The images were merged and processed using Adobe Photoshop CS3 (Adobe Systems).

### DNA Gel Blot Hybridization

Genomic DNA was digested with *Hpa*II, *Msp*I, *Nla*III or *Hae*III (New England Biolabs, USA) according to the manufacturer's instructions. Radiolabeled probes were generated by random priming, and blots were prepared and hybridized using standard methods. The following probes were generated from purified cloned inserts: 180-bp repeat (CEN) clone, pARR20-1 [37]; 45S rRNA gene clone, pARR17 [37]; and 5S rRNA gene clone, pCT4.1 [38].

### Bisulfite Sequencing

Genomic DNA samples were modified by sodium bisulfite using the EpiTech Bisulfite kit (Qiagen, USA) according to the manufacturer's protocols. PCR products were TA-cloned into pGEM-T Easy (Promega, USA) and individual clones were sequenced with the T7 primer. Approximately 24 individual clones were sequenced for each locus from two independent bisulfite sequencing experiments. Detailed bisulfite sequencing data, including the average methylation content for each clone, are provided in Table S1.

### *Hpa*II- or *Hha*I-Based Cytosine Methylation Assay

1 µg of genomic DNA was digested with *Hpa*II or *Hha*I (no enzyme for controls). Dilutions of DNA from the digestion reaction were then used for each PCR reaction. PCR conditions were 2 min at 94°C, followed by 27 cycles of 94°C for 30 s, 53°C for 30 s, and 68°C for 1 min for each primer sets.

### RT-PCR

To check the expression of the *VIM* genes, total RNA was isolated from 2-week-old leaves and inflorescence tissues from wild-type Col plants. 3-week-old leaves from each genotype were used for checking the expression of other genes. Two or three independent RNA extractions were performed per genotype and a pool of plants was used for each extraction. Aliquots of 1 µg of total RNA were treated with DNaseI (Invitrogen, USA) and 300 ng of DNase-treated total RNA was used as input in RT-PCR reactions using the Superscript III RT (Invitrogen, USA). For the 180-bp centromere repeat, we used ‘GAPC-R & CEN-F’, ‘GAPC-R & CEN-R’, or ‘no primers’ for strand-specific first-strand cDNA synthesis, and all reactions were performed with RT. The ‘no primers’ control was used to detect trace amounts of contaminating DNA, which is a particular problem due to the high-copy number of the repeat templates. For the 5S rRNA genes, we used ‘GAPC-R & 5S-R’ primers for first-strand cDNA synthesis and minus RT negative controls were performed. For other sequences, oligo(dT) primers were used for first-strand cDNA synthesis and minus RT negative controls were performed with primers specific to each sequence. Amplification of *GAPC* or *ACT2* RNA was used as an internal control. All primers used for RT PCR and the other analyses are listed in Table S2.

### Small RNA Analysis

Small RNA gel blot analysis was performed using the mirVana miRNA isolation kit (Ambion, USA) as described previously [18]. The siR1003 [39] and miR163 riboprobes were generated according to the mirVana probe construction kit (Ambion, USA) and labeled by T7 polymerase transcription in the presence of α-^32^P UTP.

## Supporting Information

Figure S1 The *VIM* genes in Arabidopsis. (A) Diagram of the domain structure of VIM proteins. All VIM proteins contain a PHD (yellow boxes) domain, two RING (blue boxes) domains, and an SRA (red boxes) domain. Each number represents the percentage amino acid sequence identity between two adjacent VIM proteins in a designated domain. A cladogram tree on the left shows the relationship among VIM proteins based on amino acid sequence identity. (B) Schematic diagram of the conserved intron/exon structure of the *VIM* genes. Boxed regions are exons and lines are introns. The positions of *vim1-2*, *vim2-2*, and *vim3 1* T-DNA insertions are marked by inverted triangles. Colored arrowheads indicate the positions of primers used for RT-PCR analysis. For *VIM1* and *VIM5*, primer sets specific for the individual genes were used. However, because of the high level of nucleotide sequence identity among *VIM2*, *VIM3*, and *VIM4*, we used two primer sets that would recognize multiple genes, and then distinguished among the products by restriction fragment length polymorphisms. We chose a primer set (*VIM2/3/4*) that would recognize *VIM2*, *VIM3*, and *VIM4*, and then digested the RT-PCR products with HincII that have a recognition site in only *VIM2* and *VIM4* products. A second primer set (*VIM2/4*) was used for *VIM2* and *VIM4*, and then HphI can digest only products derived from *VIM4*. (C) Quantification of *VIM* transcripts in leaves and inflorescence. RNA isolated from 2-week-old wild-type Col leaves or inflorescence was used for reverse transcription. Equal amounts of the RT products were used as templates for semi-quantitative RT-PCR using primers directed against *VIM* genes and *GAPC* gene. *GAPC* was used as a control. −RT, without RT; +RT, with RT; F, inflorescence; L, leaves.
(0.21 MB PDF)

Figure S2 Expression of the *VIM* genes in *vim* mutants and *vim* knock-down lines.Expression of the VIM genes in vim mutants and vim knock-down lines. Expression of the *VIM* genes were measured in 2-week-old leaves from Col wild-type, *vim1*, *vim3*, *vim1 vim3*, *vim*KD-A, and *vim*KD-B plants. Equal amounts of the first-strand cDNA were used as templates for RT-PCR using primers directed against the *VIM* genes. *ACT2* was used as a loading control. −RT, without RT; +RT, with RT.
(0.43 MB PDF)

Figure S3 Expression of the *VIM* genes in Bor-4 transgenic lines. Expression levels of the *VIM* genes were measured in 2-week-old leaves from plants with the indicated genotypes. As expected, there is no *VIM1* expression in Bor-4, but expression of *VIM1* was detected in Bor-4 plants with a *35S:YFP:VIM1* transgene. Bor-4 plants with a *35S:YFP:VIM2* or *35S:YFP:VIM3* transgene displayed significantly increased *VIM2* or *VIM3* transcript levels, respectively. Equal amounts of first-strand cDNA sample were used as templates for RT-PCR. GAPC was used as a loading control. *VIM* transcripts were detected using primers specific for either *VIM1* (left panel) or *VIM2* and *VIM3* (right panel). PCR products were digested with *Hinc*II to distinguish *VIM2* and *VIM3* as described in Figure S1C. No amplification products were detected in minus RT negative controls. +RT, with RT.
(0.29 MB PDF)

Figure S4 DNA methylation in the centromeric repeats in vim single and double mutants. Genomic DNA samples purified from plants of the indicated genotypes were digested with *HpaII* and used for DNA gel blot analysis with a radiolabeled probe for the 180-bp centromere repeats (*CEN*). The left portion of the filter hybridization data was shown in Figure 1B.
(0.89 MB PDF)

Figure S5 DNA methylation patterns monitored by *Nla*III digestion. DNA methylation was determined by DNA gel blot analysis; genomic DNA was digested with *Nla*III and blots were hybridized with radiolabeled probes corresponding to the 180-bp centromeric repeats (left), 45S rRNA genes (middle), or 5S rRNA genes (right).
(2.20 MB PDF)

Figure S6 The abundance of 5S rRNA siRNA is not significantly changed in vim mutants or *vimKD-B*. A small RNA gel blot was hybridized with a radiolabeled riboprobe (siR1003). As a loading control, the filter was rehybridized with a probe recognizing miR163. An ethidium bromide (EtBr) stained image of the samples before transfer to the membrane is shown in the bottom row.
(1.07 MB PDF)

Figure S7 Transcriptional analysis of genic regions in the *vim1 vim3* mutant and the *vim* knock-down lines. RT-PCR was carried out using RNA samples purified from plants of the indicated genotypes, with or without RT. *ACT2* and *GAPC* were used for loading controls.
(0.34 MB PDF)

Figure S8 Late flowering phenotype of *vim1 vim2 vim3* mutant plants. Wild-type Col (Col WT), *vim1 VIM2 vim3*, and *vim1 vim2 vim3* plants are shown at the same developmental age, at the initiation of flowering. Note the elongating floral inflorescence at the center of the rosette and the larger number of rosette leaves produced by the *vim1 vim2 vim3* triple mutant. The chronological ages of the plants are indicated (20 or 30 days post-germination). The plants were grown in parallel under the same environmental conditions (22°C; long-day conditions).
(1.43 MB PDF)

Table S1 Percentage of methylated cytosines in different sequence contexts within the *AtMU1*, *AtGP1*, *5S rRNA*, and *At4g31150* genes.
(0.64 MB DOC)

Table S2 Oligonucleotide primers used in this study.
(0.12 MB DOC)
