# Supplementary figures and images for: Three SRA-Domain Methylcytosine-Binding Proteins Cooperate to Maintain Global CpG Methylation and Epigenetic Silencing in Arabidopsis
Source: PLoS Genet. 2008 Aug 15;4(8):e1000156. doi: 10.1371/journal.pgen.1000156 (PMC2491724; doi:10.1371/journal.pgen.1000156)

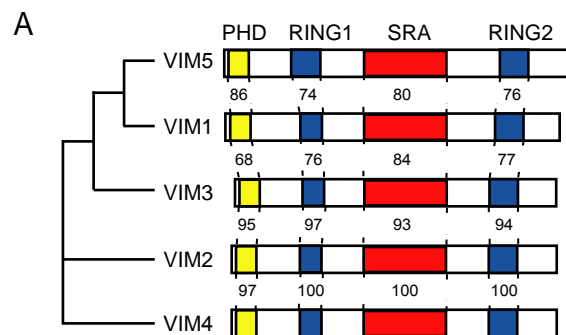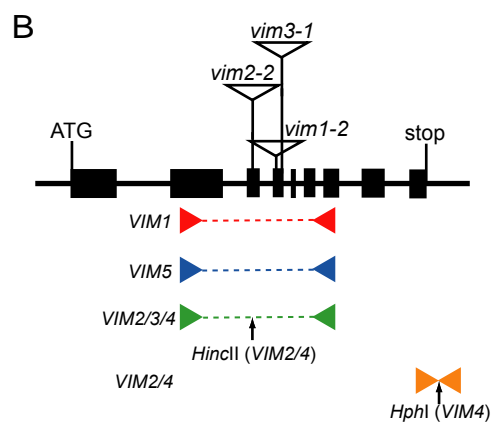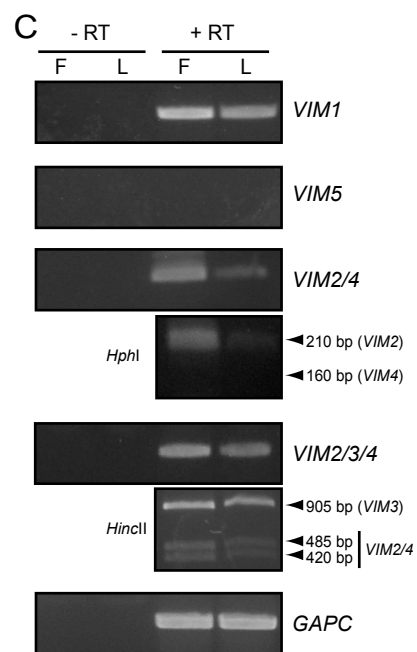

Supplement: Figure S1 — The VIM genes in Arabidopsis. (A) Diagram of the domain structure of VIM proteins. All VIM proteins contain a PHD (yellow boxes) domain, two RING (blue boxes) domains, and an SRA (red boxes) domain. Each number represents the percentage amino acid sequence identity between two adjacent VIM proteins in a designated domain. A cladogram tree on the left shows the relationship among VIM proteins based on amino acid sequence identity. (B) Schematic diagram of the conserved intron/exon structure of the VIM genes. Boxed regions are exons and lines are introns. The positions of vim1-2, vim2-2, and vim3 1 T-DNA insertions are marked by inverted triangles. Colored arrowheads indicate the positions of primers used for RT-PCR analysis. For VIM1 and VIM5, primer sets specific for the individual genes were used. However, because of the high level of nucleotide sequence identity among VIM2, VIM3, and VIM4, we used two primer sets that would recognize multiple genes, and then distinguished among the products by restriction fragment length polymorphisms. We chose a primer set (VIM2/3/4) that would recognize VIM2, VIM3, and VIM4, and then digested the RT-PCR products with HincII that have a recognition site in only VIM2 and VIM4 products. A second primer set (VIM2/4) was used for VIM2 and VIM4, and then HphI can digest only products derived from VIM4. (C) Quantification of VIM transcripts in leaves and inflorescence. RNA isolated from 2-week-old wild-type Col leaves or inflorescence was used for reverse transcription. Equal amounts of the RT products were used as templates for semi-quantitative RT-PCR using primers directed against VIM genes and GAPC gene. GAPC was used as a control. −RT, without RT; +RT, with RT; F, inflorescence; L, leaves. (0.21 MB PDF) [file pgen.1000156.s001.pdf]

Figure S2

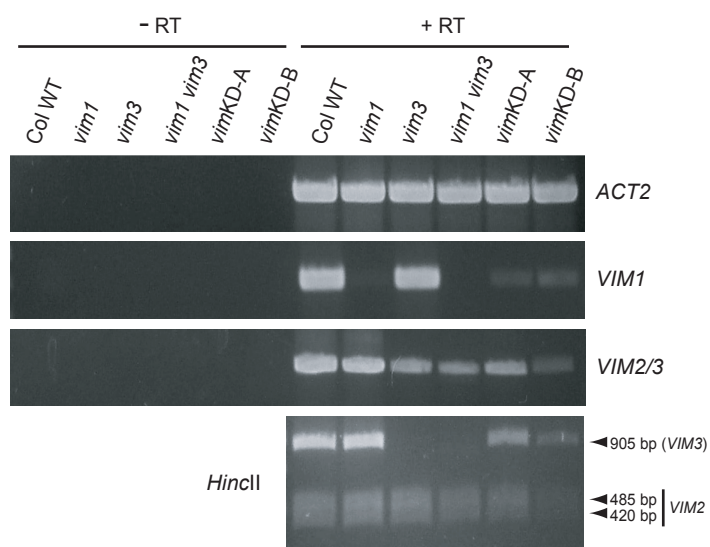

Supplement: Figure S2 — Expression of the VIM genes in vim mutants and vim knock-down lines.Expression of the VIM genes in vim mutants and vim knock-down lines. Expression of the VIM genes were measured in 2-week-old leaves from Col wild-type, vim1, vim3, vim1 vim3, vimKD-A, and vimKD-B plants. Equal amounts of the first-strand cDNA were used as templates for RT-PCR using primers directed against the VIM genes. ACT2 was used as a loading control. −RT, without RT; +RT, with RT. (0.43 MB PDF) [file pgen.1000156.s002.pdf]

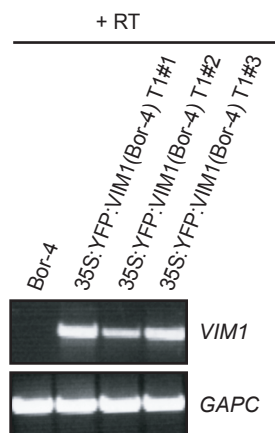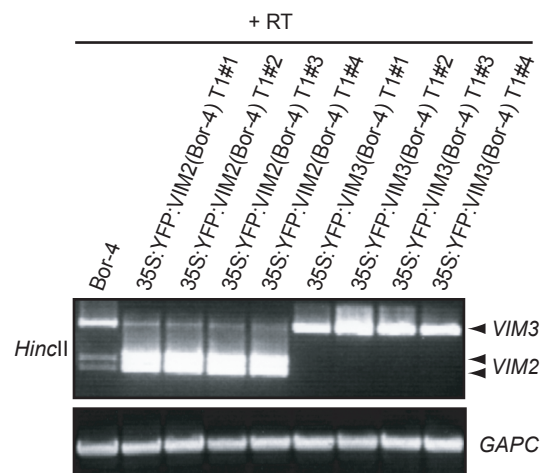

Supplement: Figure S3 — Expression of the VIM genes in Bor-4 transgenic lines. Expression levels of the VIM genes were measured in 2-week-old leaves from plants with the indicated genotypes. As expected, there is no VIM1 expression in Bor-4, but expression of VIM1 was detected in Bor-4 plants with a 35S:YFP:VIM1 transgene. Bor-4 plants with a 35S:YFP:VIM2 or 35S:YFP:VIM3 transgene displayed significantly increased VIM2 or VIM3 transcript levels, respectively. Equal amounts of first-strand cDNA sample were used as templates for RT-PCR. GAPC was used as a loading control. VIM transcripts were detected using primers specific for either VIM1 (left panel) or VIM2 and VIM3 (right panel). PCR products were digested with HincII to distinguish VIM2 and VIM3 as described in Figure S1C. No amplification products were detected in minus RT negative controls. +RT, with RT. (0.29 MB PDF) [file pgen.1000156.s003.pdf]

Figure S4

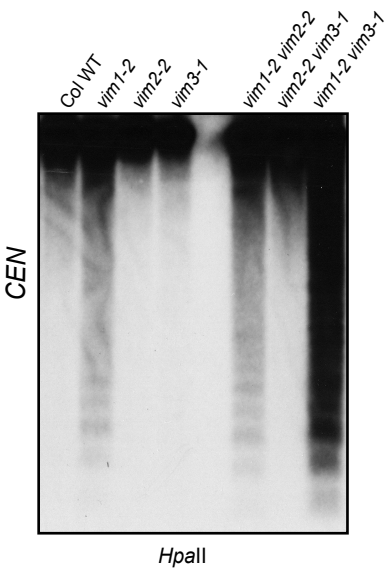

Supplement: Figure S4 — DNA methylation in the centromeric repeats in vim single and double mutants. Genomic DNA samples purified from plants of the indicated genotypes were digested with HpaII and used for DNA gel blot analysis with a radiolabeled probe for the 180-bp centromere repeats (CEN). The left portion of the filter hybridization data was shown in Figure 1B. (0.89 MB PDF) [file pgen.1000156.s004.pdf]

Figure S5

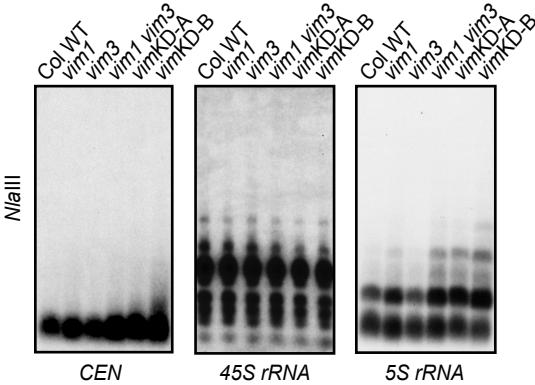

Supplement: Figure S5 — DNA methylation patterns monitored by NlaIII digestion. DNA methylation was determined by DNA gel blot analysis; genomic DNA was digested with NlaIII and blots were hybridized with radiolabeled probes corresponding to the 180-bp centromeric repeats (left), 45S rRNA genes (middle), or 5S rRNA genes (right). (2.20 MB PDF) [file pgen.1000156.s005.pdf]

Figure S6

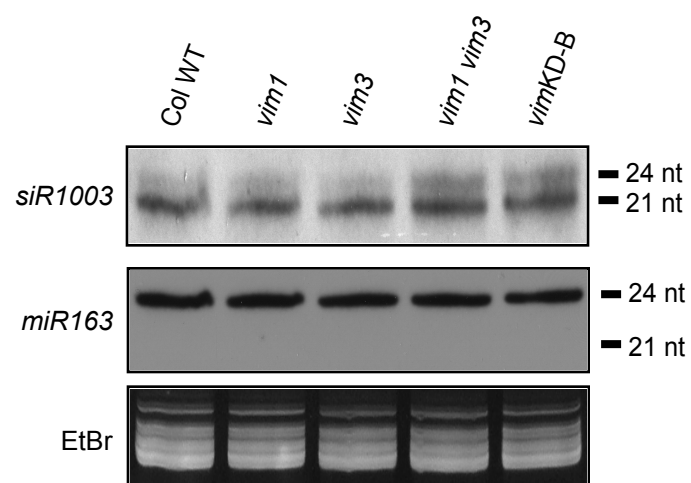

Supplement: Figure S6 — The abundance of 5S rRNA siRNA is not significantly changed in vim mutants or vimKD-B. A small RNA gel blot was hybridized with a radiolabeled riboprobe (siR1003). As a loading control, the filter was rehybridized with a probe recognizing miR163. An ethidium bromide (EtBr) stained image of the samples before transfer to the membrane is shown in the bottom row. (1.07 MB PDF) [file pgen.1000156.s006.pdf]

Figure S7

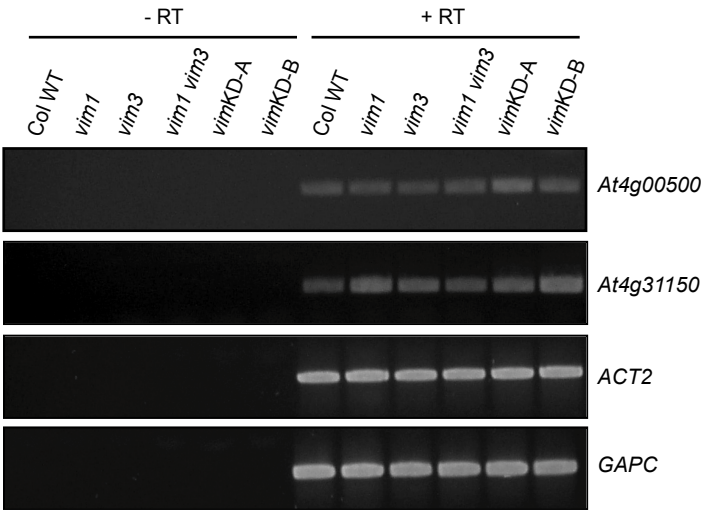

Supplement: Figure S7 — Transcriptional analysis of genic regions in the vim1 vim3 mutant and the vim knock-down lines. RT-PCR was carried out using RNA samples purified from plants of the indicated genotypes, with or without RT. ACT2 and GAPC were used for loading controls. (0.34 MB PDF) [file pgen.1000156.s007.pdf]

Figure S8

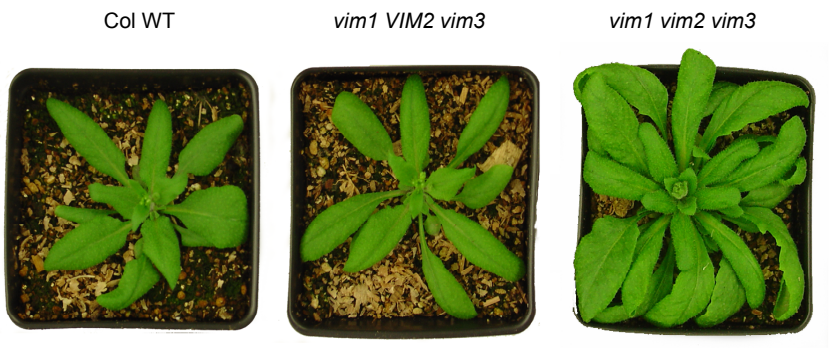

Supplement: Figure S8 — Late flowering phenotype of vim1 vim2 vim3 mutant plants. Wild-type Col (Col WT), vim1 VIM2 vim3, and vim1 vim2 vim3 plants are shown at the same developmental age, at the initiation of flowering. Note the elongating floral inflorescence at the center of the rosette and the larger number of rosette leaves produced by the vim1 vim2 vim3 triple mutant. The chronological ages of the plants are indicated (20 or 30 days post-germination). The plants were grown in parallel under the same environmental conditions (22°C; long-day conditions). (1.43 MB PDF) [file pgen.1000156.s008.pdf]
