# Supplementary material for: Three SRA-Domain Methylcytosine-Binding Proteins Cooperate to Maintain Global CpG Methylation and Epigenetic Silencing in Arabidopsis
Source: PLoS Genet. 2008 Aug 15;4(8):e1000156. doi: 10.1371/journal.pgen.1000156 (PMC2491724; doi:10.1371/journal.pgen.1000156)
Supplement: Table S1 — Percentage of methylated cytosines in different sequence contexts within the AtMU1, AtGP1, 5S rRNA, and At4g31150 genes. (0.64 MB DOC) [file pgen.1000156.s009.doc]

**Table S1.** Percentage of methylated cytosines in different sequence contexts within the *AtMU1*, *AtGP1*, *5S rRNA*, and *At4g31150* genes.

(1) *AtMU1*

Col WT *vim1 vim3*

| Clone # | CpG | CpHpG | CpHpH |  | Clone # | CpG | CpHpG | CpHpH |  | Clone # | CpG | CpHpG | CpHpH |
| --- | --- | --- | --- | --- | --- | --- | --- | --- | --- | --- | --- | --- | --- |
| 1 | 100.0% | 44.4% | 14.7% |  | 1 | 100.0% | 55.6% | 26.5% |  | 1 | 100.0% | 33.3% | 5.9% |
| 2 | 83.3% | 22.2% | 2.9% |  | 2 | 100.0% | 77.8% | 23.5% |  | 2 | 100.0% | 55.6% | 17.6% |
| 3 | 100.0% | 44.4% | 2.9% |  | 3 | 100.0% | 55.6% | 23.5% |  | 3 | 100.0% | 44.4% | 2.9% |
| 4 | 100.0% | 33.3% | 2.9% |  | 4 | 100.0% | 77.8% | 2.9% |  | 4 | 100.0% | 55.6% | 8.8% |
| 5 | 83.3% | 22.2% | 2.9% |  | 5 | 100.0% | 66.7% | 17.6% |  | 5 | 83.3% | 66.7% | 23.5% |
| 6 | 100.0% | 33.3% | 2.9% |  | 6 | 83.3% | 33.3% | 0.0% |  | 6 | 66.7% | 77.8% | 23.5% |
| 7 | 100.0% | 55.6% | 14.7% |  | 7 | 100.0% | 55.6% | 32.4% |  | 7 | 83.3% | 33.3% | 2.9% |
| 8 | 100.0% | 55.6% | 14.7% |  | 8 | 100.0% | 55.6% | 14.7% |  | 8 | 100.0% | 55.6% | 32.4% |
| 9 | 100.0% | 55.6% | 5.9% |  | 9 | 83.3% | 22.2% | 11.8% |  | 9 | 100.0% | 33.3% | 5.9% |
| 10 | 100.0% | 44.4% | 8.8% |  | 10 | 100.0% | 44.4% | 11.8% |  | 10 | 83.3% | 55.6% | 11.8% |
| 11 | 100.0% | 66.7% | 17.6% |  | 11 | 83.3% | 77.8% | 29.4% |  | 11 | 100.0% | 66.7% | 17.6% |
| 12 | 83.3% | 66.7% | 23.5% |  | 12 | 100.0% | 44.4% | 5.9% |  | 12 | 83.3% | 77.8% | 47.1% |
| 13 | 83.3% | 55.6% | 11.8% |  | 13 | 83.3% | 66.7% | 5.9% |  | 13 | 83.3% | 55.6% | 5.9% |
| 14 | 100.0% | 44.4% | 5.9% |  | 14 | 83.3% | 55.6% | 14.7% |  | 14 | 100.0% | 66.7% | 20.6% |
| 15 | 83.3% | 44.4% | 20.6% |  | 15 | 100.0% | 55.6% | 17.6% |  | 15 | 100.0% | 55.6% | 11.8% |
| 16 | 83.3% | 55.6% | 23.5% |  | 16 | 83.3% | 33.3% | 2.9% |  | 16 | 83.3% | 33.3% | 5.9% |
| 17 | 66.7% | 55.6% | 11.8% |  | 17 | 100.0% | 44.4% | 2.9% |  | 17 | 100.0% | 66.7% | 23.5% |
| 18 | 66.7% | 44.4% | 2.9% |  | 18 | 83.3% | 44.4% | 26.5% |  | 18 | 100.0% | 44.4% | 11.8% |
| 19 | 83.3% | 11.1% | 5.9% |  | 19 | 83.3% | 77.8% | 29.4% |  | 19 | 83.3% | 55.6% | 5.9% |
| 20 | 100.0% | 33.3% | 14.7% |  | 20 | 83.3% | 55.6% | 2.9% |  | 20 | 83.3% | 77.8% | 58.8% |
| 21 | 83.3% | 44.4% | 20.6% |  | 21 | 83.3% | 44.4% | 26.5% |  | 21 | 66.7% | 55.6% | 20.6% |
| 22 | 100.0% | 44.4% | 8.8% |  | 22 | 100.0% | 55.6% | 2.9% |  | 22 | 100.0% | 66.7% | 11.8% |
| 23 | 83.3% | 44.4% | 14.7% |  | 23 | 100.0% | 66.7% | 26.5% |  | 23 | 100.0% | 33.3% | 5.9% |
| 24 | 83.3% | 33.3% | 5.9% |  | 24 | 83.3% | 55.6% | 2.9% |  | 24 | 83.3% | 44.4% | 2.9% |
| AVG | 90.3% | 44.0% | 10.9% |  | AVG | 92.4% | 55.1% | 15.1% |  | AVG | 91.0% | 54.6% | 16.1% |
| SD | 10.9% | 13.7% | 7.0% |  | SD | 8.5% | 14.8% | 10.8% |  | SD | 11.0% | 14.6% | 14.0% |

*vim1 vim3* *vim*KD-A *vim*KD-B

| Clone # | CpG | CpHpG | CpHpH |  | Clone # | CpG | CpHpG | CpHpH |  | Clone # | CpG | CpHpG | CpHpH |
| --- | --- | --- | --- | --- | --- | --- | --- | --- | --- | --- | --- | --- | --- |
| 1 | 50.0% | 66.7% | 38.2% |  | 1 | 33.3% | 33.3% | 14.7% |  | 1 | 50.0% | 22.2% | 2.9% |
| 2 | 66.7% | 66.7% | 52.9% |  | 2 | 50.0% | 44.4% | 8.8% |  | 2 | 50.0% | 22.2% | 8.8% |
| 3 | 50.0% | 44.4% | 11.8% |  | 3 | 66.7% | 44.4% | 14.7% |  | 3 | 33.3% | 44.4% | 29.4% |
| 4 | 50.0% | 44.4% | 2.9% |  | 4 | 50.0% | 44.4% | 11.8% |  | 4 | 33.3% | 33.3% | 8.8% |
| 5 | 50.0% | 55.6% | 23.5% |  | 5 | 50.0% | 66.7% | 5.9% |  | 5 | 50.0% | 55.6% | 14.7% |
| 6 | 66.7% | 44.4% | 11.8% |  | 6 | 33.3% | 55.6% | 11.8% |  | 6 | 50.0% | 44.4% | 20.6% |
| 7 | 66.7% | 44.4% | 29.4% |  | 7 | 50.0% | 77.8% | 41.2% |  | 7 | 66.7% | 55.6% | 14.7% |
| 8 | 66.7% | 55.6% | 11.8% |  | 8 | 50.0% | 66.7% | 26.5% |  | 8 | 50.0% | 55.6% | 2.9% |
| 9 | 33.3% | 22.2% | 8.8% |  | 9 | 50.0% | 55.6% | 20.6% |  | 9 | 66.7% | 55.6% | 2.9% |
| 10 | 83.3% | 55.6% | 17.6% |  | 10 | 50.0% | 44.4% | 5.9% |  | 10 | 83.3% | 33.3% | 5.9% |
| 11 | 50.0% | 44.4% | 2.9% |  | 11 | 66.7% | 55.6% | 8.8% |  | 11 | 66.7% | 33.3% | 2.9% |
| 12 | 50.0% | 55.6% | 8.8% |  | 12 | 66.7% | 55.6% | 29.4% |  | 12 | 50.0% | 55.6% | 14.7% |
| 13 | 50.0% | 44.4% | 14.7% |  | 13 | 33.3% | 22.2% | 2.9% |  | 13 | 66.7% | 66.7% | 23.5% |
| 14 | 66.7% | 66.7% | 11.8% |  | 14 | 66.7% | 44.4% | 8.8% |  | 14 | 50.0% | 44.4% | 26.5% |
| 15 | 66.7% | 33.3% | 50.0% |  | 15 | 33.3% | 55.6% | 5.9% |  | 15 | 33.3% | 33.3% | 11.8% |
| 16 | 33.3% | 66.7% | 5.9% |  | 16 | 66.7% | 44.4% | 23.5% |  | 16 | 50.0% | 33.3% | 17.6% |
| 17 | 50.0% | 22.2% | 5.9% |  | 17 | 83.3% | 77.8% | 17.6% |  | 17 | 50.0% | 55.6% | 32.4% |
| 18 | 66.7% | 77.8% | 8.8% |  | 18 | 66.7% | 55.6% | 8.8% |  | 18 | 50.0% | 66.7% | 8.8% |
| 19 | 50.0% | 55.6% | 8.8% |  | 19 | 50.0% | 44.4% | 14.7% |  | 19 | 33.3% | 44.4% | 8.8% |
| 20 | 33.3% | 44.4% | 8.8% |  | 20 | 66.7% | 77.8% | 14.7% |  | 20 | 66.7% | 22.2% | 11.8% |
| 21 | 66.7% | 22.2% | 11.8% |  | 21 | 50.0% | 55.6% | 17.6% |  | 21 | 66.7% | 33.3% | 0.0% |
| 22 | 33.3% | 33.3% | 5.9% |  | 22 | 66.7% | 33.3% | 8.8% |  | 22 | 66.7% | 44.4% | 5.9% |
| 23 | 66.7% | 55.6% | 41.2% |  | 23 | 66.7% | 55.6% | 2.9% |  | 23 | 33.3% | 66.7% | 11.8% |
| 24 | 33.3% | 44.4% | 11.8% |  | 24 | 66.7% | 66.7% | 20.6% |  | 24 | 33.3% | 44.4% | 5.9% |
| AVG | 54.2% | 48.6% | 16.9% |  | AVG | 55.6% | 53.2% | 14.5% |  | AVG | 52.1% | 44.4% | 12.3% |
| SD | 14.1% | 14.9% | 14.6% |  | SD | 13.6% | 14.3% | 9.1% |  | SD | 14.2% | 13.9% | 8.9% |

(2) *AtGP1*

*Col* vim1 vim3

| Clone # | CpG | CpHpG | CpHpH |  | Clone # | CpG | CpHpG | CpHpH |  | Clone # | CpG | CpHpG | CpHpH |
| --- | --- | --- | --- | --- | --- | --- | --- | --- | --- | --- | --- | --- | --- |
| 1 | 95.0% | 40.0% | 10.0% |  | 1 | 90.0% | 63.3% | 0.0% |  | 1 | 65.0% | 40.0% | 5.0% |
| 2 | 95.0% | 60.0% | 10.0% |  | 2 | 90.0% | 53.3% | 5.0% |  | 2 | 80.0% | 70.0% | 15.0% |
| 3 | 95.0% | 46.7% | 5.0% |  | 3 | 95.0% | 56.7% | 5.0% |  | 3 | 85.0% | 53.3% | 0.0% |
| 4 | 80.0% | 43.3% | 5.0% |  | 4 | 100.0% | 60.0% | 10.0% |  | 4 | 65.0% | 40.0% | 5.0% |
| 5 | 90.0% | 60.0% | 10.0% |  | 5 | 95.0% | 53.3% | 5.0% |  | 5 | 85.0% | 50.0% | 0.0% |
| 6 | 85.0% | 43.3% | 10.0% |  | 6 | 100.0% | 66.7% | 5.0% |  | 6 | 100.0% | 73.3% | 0.0% |
| 7 | 85.0% | 43.3% | 10.0% |  | 7 | 80.0% | 50.0% | 10.0% |  | 7 | 100.0% | 66.7% | 5.0% |
| 8 | 90.0% | 76.7% | 5.0% |  | 8 | 75.0% | 56.7% | 0.0% |  | 8 | 90.0% | 56.7% | 0.0% |
| 9 | 95.0% | 43.3% | 10.0% |  | 9 | 75.0% | 50.0% | 5.0% |  | 9 | 95.0% | 53.3% | 15.0% |
| 10 | 85.0% | 70.0% | 10.0% |  | 10 | 100.0% | 83.3% | 20.0% |  | 10 | 80.0% | 63.3% | 20.0% |
| 11 | 80.0% | 40.0% | 0.0% |  | 11 | 90.0% | 76.7% | 20.0% |  | 11 | 95.0% | 53.3% | 5.0% |
| 12 | 100.0% | 83.3% | 10.0% |  | 12 | 90.0% | 50.0% | 0.0% |  | 12 | 85.0% | 46.7% | 10.0% |
| 13 | 100.0% | 70.0% | 5.0% |  | 13 | 90.0% | 56.7% | 15.0% |  | 13 | 100.0% | 80.0% | 5.0% |
| 14 | 85.0% | 63.3% | 5.0% |  | 14 | 95.0% | 70.0% | 10.0% |  | 14 | 100.0% | 56.7% | 10.0% |
| 15 | 90.0% | 56.7% | 10.0% |  | 15 | 80.0% | 60.0% | 5.0% |  | 15 | 65.0% | 36.7% | 10.0% |
| 16 | 90.0% | 30.0% | 5.0% |  | 16 | 75.0% | 50.0% | 0.0% |  | 16 | 95.0% | 70.0% | 15.0% |
| 17 | 95.0% | 60.0% | 10.0% |  | 17 | 80.0% | 50.0% | 25.0% |  | 17 | 80.0% | 70.0% | 5.0% |
| 18 | 100.0% | 56.7% | 5.0% |  | 18 | 90.0% | 56.7% | 15.0% |  | 18 | 90.0% | 46.7% | 10.0% |
| 19 | 95.0% | 40.0% | 5.0% |  | 19 | 100.0% | 86.7% | 15.0% |  | 19 | 100.0% | 53.3% | 10.0% |
| 20 | 95.0% | 40.0% | 10.0% |  | 20 | 80.0% | 56.7% | 5.0% |  | 20 | 80.0% | 43.3% | 5.0% |
| 21 | 95.0% | 76.7% | 10.0% |  | 21 | 90.0% | 76.7% | 25.0% |  | 21 | 100.0% | 53.3% | 10.0% |
| 22 | 100.0% | 66.7% | 0.0% |  | 22 | 75.0% | 56.7% | 5.0% |  | 22 | 70.0% | 53.3% | 5.0% |
| 23 | 100.0% | 76.7% | 10.0% |  | 23 | 95.0% | 60.0% | 0.0% |  | 23 | 100.0% | 80.0% | 20.0% |
| 24 | 95.0% | 36.7% | 5.0% |  | 24 | 75.0% | 63.3% | 25.0% |  | 24 |  |  |  |
| AVG | 92.3% | 55.1% | 7.3% |  | AVG | 87.7% | 61.0% | 9.6% |  | AVG | 87.2% | 57.0% | 8.0% |
| SD | 6.3% | 15.4% | 3.3% |  | SD | 9.1% | 10.7% | 8.5% |  | SD | 12.3% | 12.6% | 6.0% |

*vim1 vim3* *vim*KD-A *vim*KD-B

| Clone # | CpG | CpHpG | CpHpH |  | Clone # | CpG | CpHpG | CpHpH |  | Clone # | CpG | CpHpG | CpHpH |
| --- | --- | --- | --- | --- | --- | --- | --- | --- | --- | --- | --- | --- | --- |
| 1 | 90.0% | 63.3% | 15.0% |  | 1 | 45.0% | 56.7% | 5.0% |  | 1 | 30.0% | 46.7% | 5.0% |
| 2 | 55.0% | 43.3% | 20.0% |  | 2 | 20.0% | 46.7% | 5.0% |  | 2 | 65.0% | 70.0% | 15.0% |
| 3 | 80.0% | 70.0% | 5.0% |  | 3 | 50.0% | 56.7% | 0.0% |  | 3 | 60.0% | 70.0% | 0.0% |
| 4 | 30.0% | 50.0% | 5.0% |  | 4 | 50.0% | 43.3% | 10.0% |  | 4 | 40.0% | 40.0% | 10.0% |
| 5 | 95.0% | 66.7% | 5.0% |  | 5 | 60.0% | 63.3% | 0.0% |  | 5 | 35.0% | 36.7% | 5.0% |
| 6 | 55.0% | 70.0% | 20.0% |  | 6 | 35.0% | 66.7% | 0.0% |  | 6 | 60.0% | 53.3% | 0.0% |
| 7 | 30.0% | 60.0% | 10.0% |  | 7 | 55.0% | 53.3% | 0.0% |  | 7 | 30.0% | 36.7% | 0.0% |
| 8 | 85.0% | 83.3% | 0.0% |  | 8 | 25.0% | 50.0% | 10.0% |  | 8 | 40.0% | 63.3% | 15.0% |
| 9 | 65.0% | 60.0% | 20.0% |  | 9 | 75.0% | 50.0% | 20.0% |  | 9 | 50.0% | 63.3% | 15.0% |
| 10 | 95.0% | 83.3% | 10.0% |  | 10 | 85.0% | 66.7% | 5.0% |  | 10 | 60.0% | 46.7% | 5.0% |
| 11 | 30.0% | 40.0% | 0.0% |  | 11 | 25.0% | 43.3% | 15.0% |  | 11 | 30.0% | 40.0% | 5.0% |
| 12 | 45.0% | 66.7% | 0.0% |  | 12 | 35.0% | 50.0% | 10.0% |  | 12 | 30.0% | 63.3% | 10.0% |
| 13 | 60.0% | 43.3% | 0.0% |  | 13 | 25.0% | 53.3% | 5.0% |  | 13 | 40.0% | 56.7% | 0.0% |
| 14 | 50.0% | 40.0% | 10.0% |  | 14 | 50.0% | 76.7% | 10.0% |  | 14 | 50.0% | 46.7% | 10.0% |
| 15 | 70.0% | 73.3% | 0.0% |  | 15 | 85.0% | 76.7% | 20.0% |  | 15 | 75.0% | 50.0% | 10.0% |
| 16 | 30.0% | 40.0% | 10.0% |  | 16 | 50.0% | 60.0% | 10.0% |  | 16 | 50.0% | 66.7% | 5.0% |
| 17 | 60.0% | 43.3% | 5.0% |  | 17 | 50.0% | 43.3% | 15.0% |  | 17 | 45.0% | 66.7% | 15.0% |
| 18 | 30.0% | 46.7% | 0.0% |  | 18 | 70.0% | 73.3% | 10.0% |  | 18 | 55.0% | 50.0% | 0.0% |
| 19 | 65.0% | 40.0% | 5.0% |  | 19 | 60.0% | 40.0% | 5.0% |  | 19 | 75.0% | 66.7% | 15.0% |
| 20 | 60.0% | 53.3% | 5.0% |  | 20 | 60.0% | 53.3% | 0.0% |  | 20 | 40.0% | 43.3% | 5.0% |
| 21 |  |  |  |  | 21 | 25.0% | 46.7% | 5.0% |  | 21 | 55.0% | 50.0% | 5.0% |
| 22 |  |  |  |  | 22 | 80.0% | 76.7% | 15.0% |  | 22 |  |  |  |
| 23 |  |  |  |  | 23 | 80.0% | 83.3% | 15.0% |  | 23 |  |  |  |
| 24 |  |  |  |  | 24 |  |  |  |  | 24 |  |  |  |
| AVG | 59.0% | 56.8% | 7.3% |  | AVG | 52.0% | 57.8% | 8.3% |  | AVG | 48.3% | 53.7% | 7.1% |
| SD | 22.2% | 14.7% | 7.0% |  | SD | 20.7% | 12.8% | 6.3% |  | SD | 14.2% | 11.3% | 5.6% |

(3) *5S rRNA*

Col *vim1 vim3*

| Clone # | CpG | CpHpG | CpHpH |  | Clone # | CpG | CpHpG | CpHpH |  | Clone # | CpG | CpHpG | CpHpH |
| --- | --- | --- | --- | --- | --- | --- | --- | --- | --- | --- | --- | --- | --- |
| 1 | 100.0% | 33.3% | 11.3% |  | 1 | 73.3% | 50.0% | 22.6% |  | 1 | 66.7% | 33.3% | 7.5% |
| 2 | 93.3% | 66.7% | 15.1% |  | 2 | 73.3% | 50.0% | 39.6% |  | 2 | 86.7% | 50.0% | 22.6% |
| 3 | 80.0% | 66.7% | 1.9% |  | 3 | 46.7% | 33.3% | 1.9% |  | 3 | 66.7% | 33.3% | 7.5% |
| 4 | 100.0% | 33.3% | 11.3% |  | 4 | 93.3% | 33.3% | 13.2% |  | 4 | 60.0% | 50.0% | 20.8% |
| 5 | 100.0% | 33.3% | 1.9% |  | 5 | 60.0% | 66.7% | 30.2% |  | 5 | 73.3% | 50.0% | 24.5% |
| 6 | 93.3% | 50.0% | 11.3% |  | 6 | 86.7% | 33.3% | 9.4% |  | 6 | 100.0% | 66.7% | 20.8% |
| 7 | 93.3% | 16.7% | 5.7% |  | 7 | 60.0% | 33.3% | 7.5% |  | 7 | 100.0% | 83.3% | 26.4% |
| 8 | 86.7% | 50.0% | 5.7% |  | 8 | 86.7% | 66.7% | 22.6% |  | 8 | 80.0% | 33.3% | 28.3% |
| 9 | 86.7% | 16.7% | 1.9% |  | 9 | 66.7% | 33.3% | 11.3% |  | 9 | 100.0% | 33.3% | 15.1% |
| 10 | 93.3% | 33.3% | 7.5% |  | 10 | 86.7% | 33.3% | 7.5% |  | 10 | 86.7% | 50.0% | 22.6% |
| 11 | 100.0% | 50.0% | 5.7% |  | 11 | 86.7% | 33.3% | 15.1% |  | 11 | 66.7% | 50.0% | 15.1% |
| 12 | 100.0% | 33.3% | 13.2% |  | 12 | 93.3% | 50.0% | 3.8% |  | 12 | 86.7% | 16.7% | 15.1% |
| 13 | 93.3% | 33.3% | 1.9% |  | 13 | 93.3% | 66.7% | 9.4% |  | 13 | 100.0% | 50.0% | 13.2% |
| 14 | 100.0% | 66.7% | 5.7% |  | 14 | 80.0% | 50.0% | 24.5% |  | 14 | 100.0% | 50.0% | 5.7% |
| 15 | 93.3% | 33.3% | 7.5% |  | 15 | 66.7% | 50.0% | 15.1% |  | 15 | 86.7% | 33.3% | 18.9% |
| 16 | 100.0% | 50.0% | 5.7% |  | 16 | 86.7% | 66.7% | 9.4% |  | 16 | 93.3% | 50.0% | 30.2% |
| 17 | 86.7% | 50.0% | 22.6% |  | 17 | 80.0% | 50.0% | 15.1% |  | 17 | 93.3% | 50.0% | 15.1% |
| 18 | 86.7% | 33.3% | 22.6% |  | 18 | 73.3% | 66.7% | 24.5% |  | 18 | 66.7% | 33.3% | 18.9% |
| 19 | 100.0% | 50.0% | 5.7% |  | 19 | 93.3% | 50.0% | 13.2% |  | 19 | 100.0% | 33.3% | 5.7% |
| 20 | 100.0% | 33.3% | 13.2% |  | 20 | 66.7% | 50.0% | 1.9% |  | 20 | 66.7% | 16.7% | 3.8% |
| 21 | 93.3% | 50.0% | 13.2% |  | 21 | 86.7% | 33.3% | 5.7% |  | 21 | 86.7% | 33.3% | 24.5% |
| 22 | 66.7% | 50.0% | 3.8% |  | 22 | 80.0% | 66.7% | 13.2% |  | 22 | 86.7% | 33.3% | 20.8% |
| 23 | 93.3% | 50.0% | 18.9% |  | 23 | 66.7% | 66.7% | 26.4% |  | 23 | 86.7% | 33.3% | 5.7% |
| 24 | 80.0% | 50.0% | 5.7% |  | 24 | 86.7% | 33.3% | 22.6% |  | 24 |  |  |  |
| AVG | 92.5% | 43.1% | 9.1% |  | AVG | 78.1% | 48.6% | 15.3% |  | AVG | 84.3% | 42.0% | 16.9% |
| SD | 8.4% | 13.8% | 6.2% |  | SD | 12.7% | 13.8% | 9.6% |  | SD | 13.4% | 15.0% | 7.9% |

*vim1 vim3* *vim*KD-A *vim*KD-B

| Clone # | CpG | CpHpG | CpHpH |  | Clone # | CpG | CpHpG | CpHpH |  | Clone # | CpG | CpHpG | CpHpH |
| --- | --- | --- | --- | --- | --- | --- | --- | --- | --- | --- | --- | --- | --- |
| 1 | 46.7% | 66.7% | 52.8% |  | 1 | 53.3% | 33.3% | 11.3% |  | 1 | 33.3% | 33.3% | 28.3% |
| 2 | 46.7% | 33.3% | 9.4% |  | 2 | 53.3% | 50.0% | 37.7% |  | 2 | 46.7% | 50.0% | 54.7% |
| 3 | 66.7% | 66.7% | 24.5% |  | 3 | 80.0% | 33.3% | 32.1% |  | 3 | 80.0% | 33.3% | 35.8% |
| 4 | 40.0% | 33.3% | 9.4% |  | 4 | 53.3% | 50.0% | 39.6% |  | 4 | 86.7% | 33.3% | 66.0% |
| 5 | 40.0% | 33.3% | 35.8% |  | 5 | 53.3% | 33.3% | 35.8% |  | 5 | 46.7% | 33.3% | 26.4% |
| 6 | 33.3% | 50.0% | 11.3% |  | 6 | 53.3% | 66.7% | 41.5% |  | 6 | 66.7% | 50.0% | 30.2% |
| 7 | 60.0% | 33.3% | 9.4% |  | 7 | 53.3% | 66.7% | 32.1% |  | 7 | 60.0% | 50.0% | 52.8% |
| 8 | 60.0% | 50.0% | 20.8% |  | 8 | 46.7% | 50.0% | 28.3% |  | 8 | 33.3% | 66.7% | 35.8% |
| 9 | 46.7% | 33.3% | 9.4% |  | 9 | 80.0% | 33.3% | 7.5% |  | 9 | 46.7% | 66.7% | 45.3% |
| 10 | 60.0% | 66.7% | 54.7% |  | 10 | 73.3% | 50.0% | 11.3% |  | 10 | 60.0% | 50.0% | 28.3% |
| 11 | 40.0% | 66.7% | 39.6% |  | 11 | 73.3% | 50.0% | 37.7% |  | 11 | 53.3% | 33.3% | 47.2% |
| 12 | 40.0% | 50.0% | 52.8% |  | 12 | 73.3% | 50.0% | 41.5% |  | 12 | 33.3% | 50.0% | 66.0% |
| 13 | 73.3% | 50.0% | 15.1% |  | 13 | 80.0% | 50.0% | 30.2% |  | 13 | 40.0% | 33.3% | 22.6% |
| 14 | 40.0% | 50.0% | 37.7% |  | 14 | 53.3% | 50.0% | 7.5% |  | 14 | 40.0% | 50.0% | 60.4% |
| 15 | 46.7% | 50.0% | 9.4% |  | 15 | 73.3% | 50.0% | 11.3% |  | 15 | 53.3% | 33.3% | 41.5% |
| 16 | 73.3% | 66.7% | 9.4% |  | 16 | 66.7% | 50.0% | 17.0% |  | 16 | 86.7% | 33.3% | 22.6% |
| 17 | 66.7% | 50.0% | 37.7% |  | 17 | 46.7% | 33.3% | 15.1% |  | 17 | 53.3% | 50.0% | 47.2% |
| 18 | 73.3% | 66.7% | 13.2% |  | 18 | 80.0% | 83.3% | 50.9% |  | 18 | 40.0% | 66.7% | 26.4% |
| 19 | 60.0% | 66.7% | 26.4% |  | 19 | 46.7% | 50.0% | 17.0% |  | 19 | 40.0% | 50.0% | 20.8% |
| 20 | 53.3% | 50.0% | 37.7% |  | 20 | 66.7% | 66.7% | 49.1% |  | 20 | 73.3% | 66.7% | 37.7% |
| 21 | 46.7% | 33.3% | 30.2% |  | 21 | 46.7% | 33.3% | 26.4% |  | 21 | 46.7% | 33.3% | 28.3% |
| 22 | 73.3% | 33.3% | 11.3% |  | 22 | 46.7% | 33.3% | 17.0% |  | 22 | 86.7% | 66.7% | 24.5% |
| 23 | 60.0% | 50.0% | 26.4% |  | 23 | 53.3% | 50.0% | 30.2% |  | 23 | 40.0% | 66.7% | 49.1% |
| 24 | 66.7% | 50.0% | 28.3% |  | 24 | 66.7% | 33.3% | 52.8% |  | 24 | 73.3% | 33.3% | 30.2% |
| AVG | 54.7% | 50.0% | 25.6% |  | AVG | 61.4% | 47.9% | 28.4% |  | AVG | 55.0% | 47.2% | 38.7% |
| SD | 12.9% | 13.0% | 15.3% |  | SD | 12.6% | 13.3% | 14.1% |  | SD | 17.8% | 13.6% | 14.1% |

(4) *At4g31150*

| Clone # | CpG | CpHpG | CpHpH |  | Clone # | CpG | CpHpG | CpHpH |  | Clone # | CpG | CpHpG | CpHpH |
| --- | --- | --- | --- | --- | --- | --- | --- | --- | --- | --- | --- | --- | --- |
| 1 | 75.0% | 0.0% | 0.0% |  | 1 | 62.5% | 4.5% | 5.6% |  | 1 | 62.5% | 0.0% | 0.0% |
| 2 | 87.5% | 0.0% | 3.7% |  | 2 | 50.0% | 9.1% | 1.9% |  | 2 | 62.5% | 0.0% | 1.9% |
| 3 | 62.5% | 0.0% | 1.9% |  | 3 | 50.0% | 4.5% | 1.9% |  | 3 | 62.5% | 4.5% | 5.6% |
| 4 | 50.0% | 0.0% | 0.0% |  | 4 | 50.0% | 13.6% | 3.7% |  | 4 | 62.5% | 9.1% | 1.9% |
| 5 | 62.5% | 0.0% | 3.7% |  | 5 | 37.5% | 0.0% | 3.7% |  | 5 | 62.5% | 0.0% | 0.0% |
| 6 | 37.5% | 0.0% | 5.6% |  | 6 | 62.5% | 0.0% | 0.0% |  | 6 | 62.5% | 4.5% | 7.4% |
| 7 | 75.0% | 0.0% | 3.7% |  | 7 | 75.0% | 4.5% | 1.9% |  | 7 | 50.0% | 4.5% | 1.9% |
| 8 | 62.5% | 0.0% | 3.7% |  | 8 | 50.0% | 4.5% | 7.4% |  | 8 | 62.5% | 0.0% | 1.9% |
| 9 | 62.5% | 4.5% | 5.6% |  | 9 | 50.0% | 0.0% | 3.7% |  | 9 | 62.5% | 4.5% | 1.9% |
| 10 | 62.5% | 0.0% | 3.7% |  | 10 | 37.5% | 0.0% | 3.7% |  | 10 | 62.5% | 0.0% | 1.9% |
| 11 | 62.5% | 4.5% | 1.9% |  | 11 | 62.5% | 0.0% | 1.9% |  | 11 | 50.0% | 0.0% | 1.9% |
| 12 | 62.5% | 0.0% | 0.0% |  | 12 | 62.5% | 0.0% | 1.9% |  | 12 | 62.5% | 9.1% | 1.9% |
| 13 | 75.0% | 0.0% | 1.9% |  | 13 | 62.5% | 4.5% | 1.9% |  | 13 | 87.5% | 0.0% | 3.7% |
| 14 | 62.5% | 0.0% | 5.6% |  | 14 | 62.5% | 9.1% | 1.9% |  | 14 | 62.5% | 4.5% | 1.9% |
| 15 | 62.5% | 0.0% | 3.7% |  | 15 | 62.5% | 4.5% | 1.9% |  | 15 | 62.5% | 0.0% | 13.0% |
| 16 | 62.5% | 4.5% | 3.7% |  | 16 | 75.0% | 13.6% | 13.0% |  | 16 | 62.5% | 13.6% | 5.6% |
| 17 | 62.5% | 0.0% | 1.9% |  | 17 | 62.5% | 4.5% | 3.7% |  | 17 | 62.5% | 0.0% | 1.9% |
| 18 | 62.5% | 4.5% | 1.9% |  | 18 | 37.5% | 0.0% | 1.9% |  | 18 | 50.0% | 4.5% | 3.7% |
| 19 | 62.5% | 4.5% | 13.0% |  | 19 | 37.5% | 0.0% | 5.6% |  | 19 | 62.5% | 4.5% | 1.9% |
| 20 | 62.5% | 13.6% | 1.9% |  | 20 | 62.5% | 4.5% | 1.9% |  | 20 | 50.0% | 4.5% | 3.7% |
| 21 | 75.0% | 0.0% | 0.0% |  | 21 | 62.5% | 0.0% | 3.7% |  | 21 | 62.5% | 0.0% | 1.9% |
| 22 | 75.0% | 0.0% | 0.0% |  | 22 | 62.5% | 4.5% | 1.9% |  | 22 | 62.5% | 4.5% | 5.6% |
| 23 |  |  |  |  | 23 |  |  |  |  | 23 | 62.5% | 9.1% | 3.7% |
| 24 |  |  |  |  | 24 |  |  |  |  | 24 |  |  |  |
| AVG | 64.8% | 1.7% | 3.0% |  | AVG | 56.3% | 3.9% | 3.4% |  | AVG | 61.4% | 3.6% | 3.2% |
| SD | 9.9% | 3.3% | 2.9% |  | SD | 11.4% | 4.3% | 2.7% |  | SD | 7.5% | 3.9% | 2.8% |

Col *vim1 vim3*

*vim1 vim3* *vim*KD-A *vim*KD-B

| Clone # | CpG | CpHpG | CpHpH |  | Clone # | CpG | CpHpG | CpHpH |  | Clone # | CpG | CpHpG | CpHpH |
| --- | --- | --- | --- | --- | --- | --- | --- | --- | --- | --- | --- | --- | --- |
| 1 | 12.5% | 0.0% | 1.9% |  | 1 | 37.5% | 0.0% | 5.6% |  | 1 | 25.0% | 0.0% | 3.7% |
| 2 | 25.0% | 0.0% | 1.9% |  | 2 | 25.0% | 0.0% | 3.7% |  | 2 | 25.0% | 4.5% | 5.6% |
| 3 | 25.0% | 0.0% | 3.7% |  | 3 | 37.5% | 4.5% | 1.9% |  | 3 | 25.0% | 4.5% | 7.4% |
| 4 | 37.5% | 4.5% | 3.7% |  | 4 | 37.5% | 0.0% | 3.7% |  | 4 | 25.0% | 4.5% | 0.0% |
| 5 | 25.0% | 0.0% | 7.4% |  | 5 | 12.5% | 9.1% | 3.7% |  | 5 | 12.5% | 0.0% | 3.7% |
| 6 | 25.0% | 4.5% | 0.0% |  | 6 | 25.0% | 4.5% | 1.9% |  | 6 | 0.0% | 0.0% | 7.4% |
| 7 | 12.5% | 0.0% | 0.0% |  | 7 | 50.0% | 9.1% | 5.6% |  | 7 | 12.5% | 0.0% | 0.0% |
| 8 | 37.5% | 4.5% | 1.9% |  | 8 | 12.5% | 4.5% | 0.0% |  | 8 | 37.5% | 4.5% | 3.7% |
| 9 | 37.5% | 0.0% | 5.6% |  | 9 | 0.0% | 4.5% | 1.9% |  | 9 | 0.0% | 0.0% | 3.7% |
| 10 | 12.5% | 0.0% | 1.9% |  | 10 | 37.5% | 0.0% | 1.9% |  | 10 | 25.0% | 0.0% | 0.0% |
| 11 | 0.0% | 4.5% | 0.0% |  | 11 | 37.5% | 0.0% | 0.0% |  | 11 | 37.5% | 0.0% | 3.7% |
| 12 | 37.5% | 13.6% | 1.9% |  | 12 | 37.5% | 0.0% | 3.7% |  | 12 | 0.0% | 0.0% | 3.7% |
| 13 | 25.0% | 4.5% | 3.7% |  | 13 | 37.5% | 0.0% | 1.9% |  | 13 | 12.5% | 0.0% | 0.0% |
| 14 | 37.5% | 0.0% | 3.7% |  | 14 | 50.0% | 4.5% | 3.7% |  | 14 | 0.0% | 0.0% | 3.7% |
| 15 | 50.0% | 0.0% | 0.0% |  | 15 | 75.0% | 4.5% | 1.9% |  | 15 | 12.5% | 0.0% | 1.9% |
| 16 | 50.0% | 4.5% | 1.9% |  | 16 | 25.0% | 0.0% | 1.9% |  | 16 | 37.5% | 4.5% | 5.6% |
| 17 | 0.0% | 4.5% | 3.7% |  | 17 | 25.0% | 0.0% | 3.7% |  | 17 | 12.5% | 9.1% | 3.7% |
| 18 | 12.5% | 0.0% | 1.9% |  | 18 | 25.0% | 0.0% | 3.7% |  | 18 | 25.0% | 0.0% | 0.0% |
| 19 | 25.0% | 0.0% | 1.9% |  | 19 | 12.5% | 0.0% | 0.0% |  | 19 | 37.5% | 4.5% | 3.7% |
| 20 | 50.0% | 0.0% | 1.9% |  | 20 | 37.5% | 0.0% | 5.6% |  | 20 | 25.0% | 9.1% | 0.0% |
| 21 |  |  |  |  | 21 | 25.0% | 0.0% | 1.9% |  | 21 | 50.0% | 0.0% | 1.9% |
| 22 |  |  |  |  | 22 | 12.5% | 0.0% | 5.6% |  | 22 | 25.0% | 0.0% | 0.0% |
| 23 |  |  |  |  | 23 | 25.0% | 0.0% | 1.9% |  | 23 | 25.0% | 4.5% | 0.0% |
| 24 |  |  |  |  | 24 | 25.0% | 0.0% | 3.7% |  | 24 | 0.0% | 4.5% | 0.0% |
| AVG | 26.9% | 2.3% | 2.4% |  | AVG | 30.2% | 1.9% | 2.9% |  | AVG | 20.3% | 2.3% | 2.6% |
| SD | 15.3% | 3.5% | 1.9% |  | SD | 15.6% | 3.0% | 1.7% |  | SD | 14.2% | 3.0% | 2.4% |
