# Supplementary material for: Three SRA-Domain Methylcytosine-Binding Proteins Cooperate to Maintain Global CpG Methylation and Epigenetic Silencing in Arabidopsis
Source: PLoS Genet. 2008 Aug 15;4(8):e1000156. doi: 10.1371/journal.pgen.1000156 (PMC2491724; doi:10.1371/journal.pgen.1000156)
Supplement: Table S2 — Oligonucleotide primers used in this study. (0.12 MB DOC) [file pgen.1000156.s010.doc]

**Table S2. Oligonucleotide primers used in this study.**

| **Name** | **Sequence (5’🡪3’)** | **Description** | **Reference** |
| --- | --- | --- | --- |
| VIM1-F | GCAGGGAAAGAGAACTTGTGGTAA | RT-PCR | This study |
| VIM1-R | AAACATTCACTGACTTTTTACTT |
| VIM4-F | GCAGGGACACCGCAGTTGTGGAAC | RT-PCR | This study |
| VIM4-R | ACATTTTTAACTGCCTTTTTACTA |
| VIM2/3/5-F | AAGCAAAATTCCGAGACATGTGGC | RT-PCR | This study |
| VIM2/3/5-R | ATCCAAAGCCATTCTGCTAACA |
| VIM2/5-F | tgctgcacaaccgatctctccgag | RT-PCR | This study |
| VIM2/5-R | gaaaaactaaagagctccttaagc |
| GAPC-F | CTGTCAACGACCCCTTCATC | RT-PCR | [1] |
| GAPC-R | CCTGTTGTCGCCACCGAAGTC |
| ACT2-F | TGATATTCAACCCAATCGTGTGTGAC | RT-PCR | This study |
| ACT2-R | AAGCAAGAATGGAACCACCGATCC |
| VIM1FL-F | caccatggcgcgtgacatgcaactc | cDNA subcloning | This study |
| VIM1FL-R | tcacctcatggtcgcagaaactgt |
| VIM2FL-F | caccatggcgattcaaactcaggtt | cDNA subcloning | This study |
| VIM2FL-R | ttaagctgagagacttgtaccacc |
| VIM3FL-F | caccatggcgattgaaactcagctt | cDNA subcloning | This study |
| VIM3FL-R | ttaagctgagagacttgtaccact |
| AtMU1BI-F | AGGGGAYYTAAGTATTGGTTGAAT | Bisulfite sequencing | This study |
| AtMU1BI-R | CCTTCTTARCCTTCTTTTCAATCT |
| AtGP1BI-F | GGAGTGGGGGYTGGTGGAGYTGGT | Bisulfite sequencing | This study |
| AtGP1BI-R | TCCCCCTCTARAAAATRCACTRTT |
| 5SBI-F | GGYATTTTYGTGATTTGGGYTATA | Bisulfite sequencing | This study |
| 5SBI-R | TATCACATRCCAARTTTRRCCTCAC |
| ROS1-F | AGAAGAAATTCCTACCATCA | RT-PCR | [2] |
| ROS1-R | ACCGTTCTTCGAGGTAATTC |
| At4g00500-F | TGAGTTTGAGCCTATCCCTAGGAT | *Hpa*II-PCR; RT-PCR | This study |
| At4g00500-R | CCTCTTTCTCTCAATGTTCCCCAA |
| At4g13610-F | CAAATGGGATTGTGAAAAACATTG | *Hpa*II-PCR; RT-PCR | This study |
| At4g13610-R | TCCTAAGGTGTAGGGCTTCTTTAA |
| At4g23560-F | CCCTGGTTTACACATTCTAATAGT | *Hpa*II-PCR | This study |
| At4g23560-R | ATAAAGCTACGTTAGATCACGATT |
| At4g31150-F | ATAAAGCTACGTTAGATCACGATT | *Hpa*II-PCR | This study |
| At4g31150-R | ACAAAAAAGGAAGCAAAGAGACAT |
| At4g31150BI-F | TTTTARCAACRAAACTTCTCCATA | Bisulfite sequencing | This study |
| At4g31150BI-R | GAYAATGGTTAGAAATGGYGGAGA |

**Table S2 (continued).** Oligonucleotide primers used in this study.

| **Name** | **Sequence (5’🡪3’)** | **Description** | **Reference** |
| --- | --- | --- | --- |
| CEN-F | accatcaaagccttgagaagca | RT-PCR | [3] |
| CEN-R | ccgtatcactgtttctgtttctatgttgt |
| 5S-F | GGATGCGATCATACCAG | RT-PCR | [4] |
| 5S-R | CGAAAAGGTATCACATGCC |
| AtMU1-F | GTGGATATACCAAAAACACAA | RT-PCR | [5] |
| AtMU1-R | CTTAGCCTTCTTTTCAATCTCA |
| AtLINE1-4-F | CCGATGGTGACCAAGAGTTT | RT-PCR | [5] |
| AtLINE1-4-R | TCAATGTCGGAGACCTCCTC |
| AtGP1-F | ACAGTGCCACAGTTGAGCAG | RT-PCR | [5] |
| AtGP1-F | CAGAAAAATACTCGGTGCCAAT |
| FWA-F | TTAGTAAAGAATCAATTGGGTTTA | *Hha*I-PCR | This study |
| FWA-R | GAACCAAAATCATTCTCTAAACAA |
| FWART-F | agaacccataggcgcactgcttat | RT-PCR | This study |
| FWART-R | cagacgtcctagttccaccactaa |
| CMT3-F | CTCGATGCCATTATAGACGTGC | RT-PCR | This study |
| CMT3-R | GTCTCTGGGTGGTTATGCTGC |
| DRM2-F | AGCGATCTATGTGTAATGAGTCCG | RT-PCR | This study |
| DRM2-R | GCAAAATACGGTCTTTGGTGAAG |
| MET1-F | AAGTTGAAAATCTCACTATCTC | RT-PCR | This study |
| MET1-R | TGCCAAATTTGGGTGGTTATC |
| DME-F | [TGAGAGGAAGTTTTCCGCTC](http://www.idtdna.com/OrderStatus/                                                                SpecSheet.aspx?OrderNum=4658643&MfgID=36331640&MfgLocID=1&SearchDays=&SearchNum=&SearchPO=&SearchRef=&ProdID=1213) | RT-PCR | [2] |
| DME-R | [GTGTCGCTTCTGCTCTATGA](http://www.idtdna.com/OrderStatus/                                                                SpecSheet.aspx?OrderNum=4658643&MfgID=36331641&MfgLocID=1&SearchDays=&SearchNum=&SearchPO=&SearchRef=&ProdID=1213) |
| At4g31150RT-F | CTGCATCATGTGGATGGTCTGAA | RT-PCR | This study |
| At4g31150RT-R | TAAAGACTTTGGAGAGTTTCTAT |

**References**

1. Kerschen A, Napoli CA, Jorgensen RA, Muller AE (2004) Effectiveness of RNA interference in transgenic plants. FEBS Lett 566: 223-228.

2. Mathieu O, Reinders J, Caikovski M, Smathajitt C, Paszkowski J (2007) Transgenerational stability of the Arabidopsis epigenome is coordinated by CG methylation. Cell 130: 851-862.

3. May BP, Lippman ZB, Fang Y, Spector DL, Martienssen RA (2005) Differential regulation of strand-specific transcripts from Arabidopsis centromeric satellite repeats. PLoS Genet 1: e79.

4. Vaillant I, Schubert I, Tourmente S, Mathieu O (2006) MOM1 mediates DNA-methylation-independent silencing of repetitive sequences in Arabidopsis. EMBO Rep 7: 1273-1278.

5. Zhu J, Kapoor A, Sridhar VV, Agius F, Zhu JK (2007) The DNA glycosylase/lyase ROS1 functions in pruning DNA methylation patterns in Arabidopsis. Curr Biol 17: 54-59.
